# Self-Organization of Porphyrin–POM Dyads: Nonplanar Diacids and Oxoanions in Low-Dimensional H-Bonding Networks

**DOI:** 10.3390/molecules27207060

**Published:** 2022-10-19

**Authors:** Christopher J. Kingsbury, Marc Kielmann, Brendan Twamley, Mathias O. Senge

**Affiliations:** 1 Trinity Biomedical Sciences Institute, School of Chemistry, Chair of Organic Chemistry, Trinity College Dublin, The University of Dublin, 152-160 Pearse St., 2 Dublin, Ireland; 2 School of Chemistry, Trinity College Dublin, The University of Dublin, 2 Dublin, Ireland; 3 Focus Group—Molecular and Interfacial Engineering of Organic Nanosystems, Institute for Advanced Study (TUM-IAS), Technical University of Munich, Lichtenberg-Str. 2a, 85748 Garching, Germany

**Keywords:** supramolecular chemistry, porphyrins, crystallography, polyoxometalates (POMs), molecular engineering

## Abstract

Coordinating the spatial arrangement of electroactive partners is crucial to designable molecular electronics and photonics. Porphyrins are ubiquitous reaction centers in nature; synthetic porphyrins, in the crystallographic solid state, are often coerced into monolithic stacks, inhibiting reactivity. Using the principles of self-organization, and by exploiting charge-balance principles, we can manipulate nonplanar porphyrins into one- and two-dimensional hydrogen-bonded polymers, with polyoxometalate (POM) and bifunctional counter-anions serving as linkers. Herein, we report 11 crystal structures as a systematic study of the interactions between dodecasubstituted porphyrin acids and nonstandard counterions, as well as the induced conformations in the porphyrin core. We can show that this hydrogen bond chelate is a viable method of organizing electroactive centers into filaments and monolayers for surface deposition and ultrathin devices.

## 1. Introduction

Porphyrins are widely utilized chemical and biological moieties, owing to a confluence of highly desirable properties, namely primarily visible-light interactions and facile modification by core and periphery modification [1]. As a readily synthetically accessible macrocycle [2] over 9000 crystal structures of porphyrinoid small molecules have been reported [3,4], with a further 13,000 examples in the Protein Data Bank [5], placing it among the most populous categories of structural data available. As shown previously, the shape accessed by the porphyrin is important to consider when assessing properties [6,7,8]; the ability to engineer, or to rationally design a particular conformation or arrangement is particularly enticing [9].

Porphyrin involvement in crystal engineering—using crystal organizational principles to engender a desired solid state or set of properties—is usually the domain of metalloporphyrins, owing to the macrocycle’s well-known metal chelation [10]. Metalloporphyrins are among the earliest examples of rationally designed coordination polymers [11,12], providing ‘open’ axial metal coordination sites in porous materials; these compounds are principally constructed from meso-4-carboxyphenyl- and -4-pyridylporphyrins and closely related ligands [13]. Aside from rationally designed materials, the organization of planar porphyrin compounds in the solid state is similar to that of other mostly planar organic molecules, with usual edge-to-face and face-to-face aromatic stacking predominating in lieu of hydrogen bonding [14,15,16]. Some additional motifs, such as charge-assisted C–H⋯O interactions from the porphyrin periphery, have been identified in the solid state [17]. Striking examples of supramolecular assemblies involving porphyrins have been reported by exploiting hydrogen bonding and ionic interactions at the porphyrin periphery. Porphyrin–pyridinium polycations form complexes with calixarene polyanionic cradles [18,19], as do sulfonate-decorated anionic porphyrins with cationic partners [20,21,22]. Weak interactions can similarly control relative orientation in binary systems [23,24]. These areas have been reviewed previously [25,26,27].

**Scheme 1:**
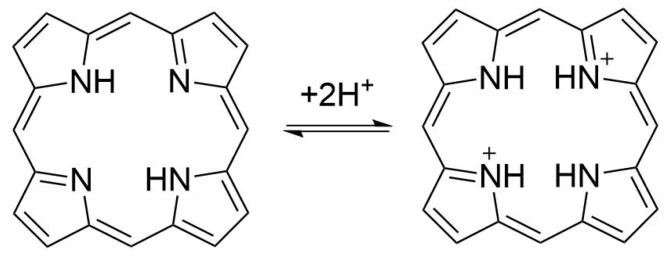
Acidification of the porphyrin core.

The predictable nature of the porphyrin core transformation upon acidification can provide characteristic H-bond chelate interactions, shown in Scheme 1, though this has been only investigated recently as a motif for forming designed supramolecular oligomeric [28] or polymeric materials [29]. Fischer’s early porphyrin studies established a transformation which occurred upon the addition of sufficiently strong acid to porphyrins—the generation of a diacid species, with a corresponding change to the absorption spectra [30]. Fleischer’s early crystallographic studies [31] identified the significant ‘saddle’-shaped structural change which occurred upon acidification, in which the porphyrin becomes nonplanar, furnishing a strong hydrogen bonding chelate from the core of the porphyrin to the counter-anion, as indicated in Scheme 1. Aside from two counterexamples of “cis-” chelates [17,32], this projective saddle motif is shared for all porphyrin diacid crystal structures [33].

**Figure 1:**
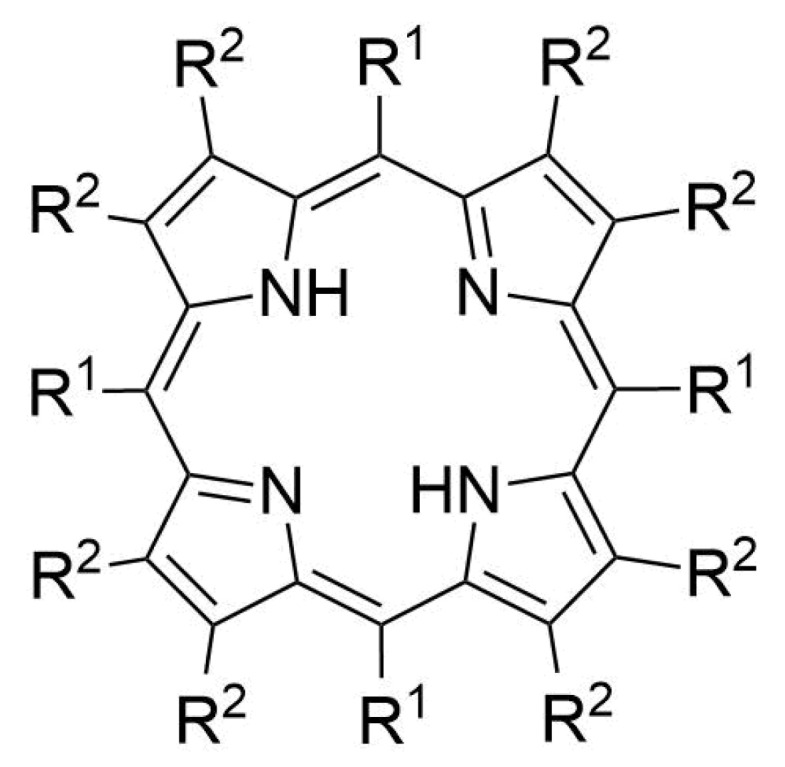
The dodecasubstituted porphyrins 2,3,7,8,12,13,17,18-octaethyl-5,10,15,20-tetraphenylporphyrin (H_2_OETPP, H_2_**1**, R^1^ = Ph, R^2^ = Et) and 2,3,7,8,12,13,17,18-octaisobutyl-5,10,15,20-tetraphenylporphyrin (H_2_OiBuTPP, H_2_**2**, R^1^ = Ph, R^2^ = iBu) discussed in this article.

Usually, a protonating agent of some strength is required to enact the transformation of a planar porphyrin to the nonplanar dicationic species. Dodecasubstituted porphyrins, e.g., H_2_OETPP (H_2_**1**, Figure 1, R^1^ = Ph, R^2^ = Et) already conform to a related saddle shape; these compounds exhibit increased basicity and are, therefore, more easily transformed to cationic species [33,34]. The complementarity of the peripheral crowding saddle shape and acidified core structural modifications allows dodecasubstituted porphyrins to potentially form H-bond adducts with a wider range of analytes than, e.g., 5,10,15-20-tetraphenylporphyrin (TPP, Figure 1, R^1^ = Ph, R^2^ = H). Due to the additional affinity required to enact the nonplanar transformation, these weaker acids would be ineffectual in transforming a normally planar free-base porphyrin. As expected, much of the available structural information for porphyrin diacids to date has been gathered only on a small collection of simple counter-anions, principally Cl^−^, ClO_4_^−^, and CF_3_COO^−^, although interactions with polyoxometalate (POM) species are known [35,36]. The methods of forming POM–porphyrin hybrids have been recently reviewed [37].

We have combined nonplanar porphyrins with a diverse collection of counter-anions beyond those commonly encountered, namely simple and complex oxyanions and various carboxylates. Our expectation was that the cation and anion would form the close associations necessary for charge transfer (e.g., proton-coupled electron transfer) [38] and could be coerced to form polymeric supramolecular interactions by this method. An investigation of the degree to which these interactions can be harnessed to provide tailored materials is certainly warranted, given the immense interest in the charge transfer between antennae (chlorophyll) and defined metal–oxo clusters (Mn_4_CaO_x_) in biological photosystems [39].

The present study provides some additional insight into the inner-core binding motif of these acid porphyrins, by providing multiple examples of porphyrin compounds with redox non-innocent partners. Similarly, the ability to form directional and ordered spatial arrangements of porphyrin motifs has significant implications for designed 2D electronic materials. The incorporation of a neutral (solvate) and charged (anion) species in a direct interaction with a highly distorted core demonstrates the promise of these materials for catalysis; the implications of this binding style are discussed.

## 2. Results

The 11 crystal structures listed in Table 1 were determined and used as a comparative set for interactions of cationic, nonplanar porphyrins with nonstandard anions, as compared to the usual counter-anions which have been reported previously. The crystallization of these compounds was concomitant with the acidification process; the use of nonplanar porphyrins allows a wider variety of acids to be used due to the increased basicity of these species, and a smaller rearrangement upon acidification.

### 2.1. The Crystal Structure H_4_OiBuTPP(HSO_4_)_2_(H_2_O)(MeOH)_3_

**Figure 2:**
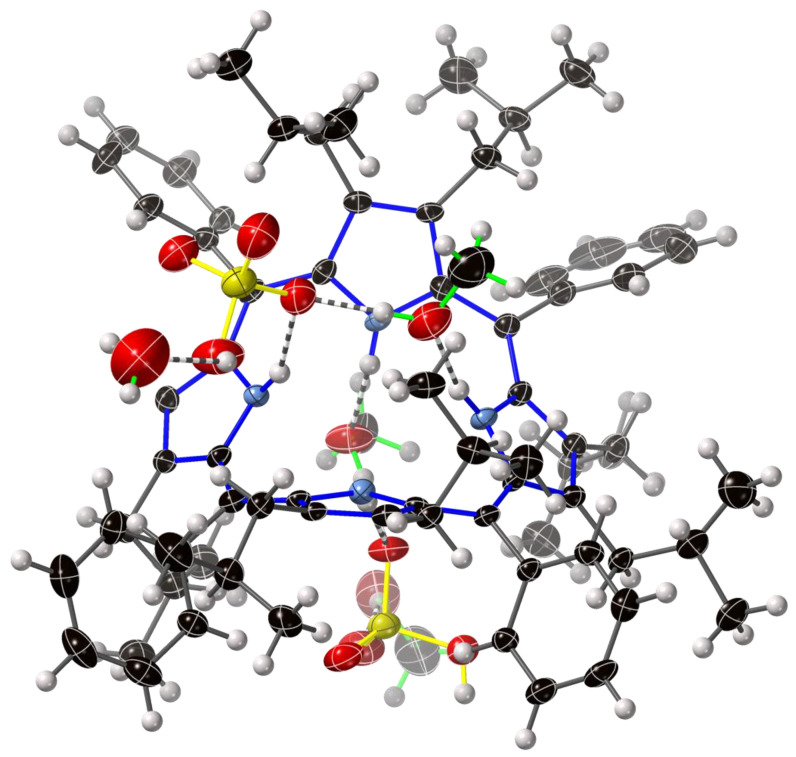
View of the molecular structure of H_4_OiBuTPP(HSO_4_)_2_(H_2_O)(MeOH)_3_ in the crystal (compound **1**). The porphyrin core (blue bonds) is distorted such that the N–H moieties can form hydrogen bonds with solvates (green) and bisulfate anions (yellow). Ellipsoids are shown at 50% probability, minor components of structural disorder are omitted, and H atoms are shown as spheres of a fixed radius.

The crystal structure of H_4_OiBuTPP(HSO_4_)_2_(H_2_O)(MeOH)_3_ (compound **1**) is shown in Figure 2 and Figure S1.1 (see Supplementary Materials). The core of this porphyrin is strongly distorted from a planar arrangement and adopts a saddle shape, consistent with previous examples of dodecasubstituted porphyrins and diacid species [7]. The N–H units in the core of the porphyrin are directed up and down from the mean plane of the porphyrin and interact with bisulfate anions and methanol solvate. The H atom placement within the N–H unit is confirmed by C_a_–N–C_a_ angles at 110.3(3)°.

The H-bonds from the porphyrin core (see Table 2) are consistent with charge-assisted conventional H-bond interactions and are shorter than those for the core-solvate (i.e., porphyrin N–H⋯O) and solvate–anion (O–H⋯O) interactions observed in this structure, but longer than anion–anion “symmetrical” H-bonds. Anion–anion H-bonds, present in only one of the two distinct bisulfate anions, arrange these molecules into weak dimer pairs of porphyrins in the solid state.

Alkyl groups (isobutyl at the 2,3,7,8,12,13,17,18-positions) demonstrate two different orientations, namely a pseudo-equatorial and a pseudo-axial projection of the CH(CH_3_)_2_ atoms with respect to the mean porphyrin plane, with these projections alternating in each pyrrole unit. Some minor disorder is observed in *i*Bu groups, as shown in Figure S9.2, with large-volume ellipsoidal tensors apparent. This arrangement is distinct from previous examples of H_2_OETPP (H_2_**1**, Figure 1), in which the dominant arrangement generally projects ethyl groups along the *S*_4_ axis of the distorted porphyrin.

A normal-coordinate structural decomposition (NSD) profile of the porphyrin core (see Table 3) allows numerical interrogation of individual macrocycle distortion from a model compound and from *D*_4*h*_ symmetry. The measured B_2u_(1) “*saddle*” and B_2u_(2) values are consistent with previous examples of H_4_-dodecasubstituted porphyrin diacids, with minor additional “*ruffle*” distortion (B_1u_) which is common for dodecasubstituted compounds. The NSD values are discussed in further detail below.

This first exemplar crystal structure displays both a macrocyclic conformation and interaction with anion and solvent characteristic of each of the compounds presented here. A further 10 diffraction patterns were measured to elucidate any trends which may be observed from the analysis of a large data set. Nuances apparent in each of the individual structures are demonstrated in supplementary images attached to this report, with general trends expanded upon below.

### 2.2. Crystal Structures with Polyanionic Partners

**Figure 3:**
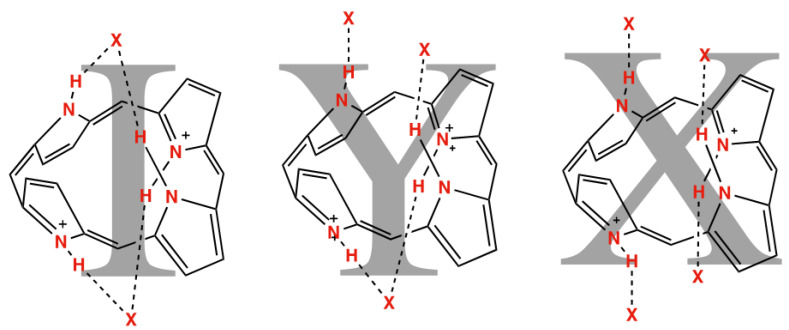
“I”, “Y”, and “X” shapes adopted by porphyrin dications interacting with two, three, and four atoms, respectively.

As has been previously established, porphyrin dication macrocycles are liable to form “pincer”-type convergent hydrogen bonding arrangements with two anions, termed an “I” shape (Figure 3) [40,41]. The addition of a hydrogen bond-forming solvate can allow for “Y” and “X” shapes of the resulting charge-balanced cluster, in which the porphyrin is able to interact with three or four distinct molecular species, respectively. These separate species are frequently linked by X–H⋯X hydrogen bonds, not indicated in this diagram, which institute a minimum foci separation of around 2.5 Å. Relevant porphyrin core hydrogen bonding distances are shown in Table 2.

**Table 2:** The H-bonding distances D⋯A of the core atoms in the discussed crystal structures.

Compound Number	Compound	N^21^–H⋯X (Å)	N^22^–H⋯X (Å)	N^23^–H⋯X (Å)	N^24^–H⋯X (Å)
**1**	[H_4_**2**](HSO_4_)_2_·H_2_O·3(MeOH)	2.775(6)	2.822(6)	2.758(9) *	2.728(6)
**2**	[H_4_**2**](O_2_C(CF_2_)_4_CO_2_)·1.5(CH_2_Cl_2_)·1.5(CH_3_OH)	2.813(2)	2.710(3)	2.879(2)	2.663(3)
**3**	[H_4_**2**](Br)_2_·1.6(CH_3_OH)	3.376(2) ^†^	3.332(2) ^†^	=N^21^	=N^22^
**4**(**1**)	[H_4_**2**]_3_(Mo^VI^_12_O_36_(PO_4_))_2_·60(CH_3_OH)·1.5(H_2_O)	2.73(2)	2.917(18)	2.912(18)	2.72(2)
**4**(**2**)		2.919(10) *	-	2.715(19)	2.978(17)
**4**(**3**)		2.914(17)	2.718(16)	-	2.899(16)
**4**(**4**)		2.71(2)	2.964(18)	2.910(13)	2.72(2)
**4**(**5**)		3.062(18)	2.882(19)	2.77(3)	2.74(2)
**4**(**6**)		2.80(2)	2.76(3)	2.75(3)	2.812(12)
**5**	[H_4_**1**](O_2_C(C(CH_2_)_3_C)CO_2_)·(HO_2_C(C(CH_2_)_3_C)CO_2_H)	2.789(6)	=N^21^	=N^21^	=N^21^
**6**	[H_4_**1**](HSe^IV^O_3_)_2_·(CH_3_OSe^IV^O_2_H)·3(CH_3_OH)	2.799(3)	2.812(4)	2.819(3)	2.768(4)
**7**	[H_4_**1**](O_2_C(CH_2_)_10_CO_2_)·4(CH_3_OH)·H_2_O	2.696(4)	2.687(4)	2.684(4)	2.677(4)
**8**	[H_4_**2**](O_2_C(CH_2_)CO_2_H)Cl_0.7_Br_0.3_·2(CH_3_OH)	2.693(5) *	3.270(4) ^†^	2.918(4) *	3.220(6) ^†^
**9**	[H_3_**2**]_2_(Mo^VI^_4_O_12_(MeO)_2_)·6(CH_3_OH)	2.895(7) *	-	3.176(6)	2.94(3) *
**10**	[H_3_**2**](O_2_CCH_3_)·0.31(H_2_O)·1.25(CH_2_Cl_2_)·0.69(CH_3_OH)	2.816(4) *	2.96(2)	=N^21^	N^24^⋯H-X
**11**	[H_4_**2**](O_2_CC_6_H_4_CO_2_H)_2_·2.5(CH_3_OH)·0.55(CH_2_Cl_2_) 0.6(H_2_O)	2.624(3)	2.890(3)	2.837(3)	2.824(3)

X = O except ^†^ X = Br; * primary component of disordered arrangement.

A porphyrindiium bis(anion) compound, such as [H_4_**2**](Br)_2_·1.6(CH_3_OH) (compound **3**), is the simplest example in the series of 22*H*,24*H*-porphyrindiium^2+^ associations with oppositely charged species, forming an “I” shape from the apparent convergent H-bonding pincer. Using a 1:2 ratio of a single porphyrin dication with two equivalents of monoanion, one would expect minimal long-range interactions which are engaged by these molecules. When a dianion, such as a hydrocarbon oligomer bis(acid) or dianionic polyoxometalate is added, a 1:1 ratio of tectons is formed; logically, this would lead to oligomers or one-dimensional polymers in the solid state.

Using a charge-balance concept, we can understand these observations using A. F. Wells’ enumeration of nets [42]—namely that an (m,n)-net, for a 2+ connector, must use an anion partner with *n*^−^ charge. This is exemplified in the (6,3)-net structure, commonly referred to as a hexagonal tiling or “chicken-wire”, shown for the combination of [H_4_OiBuTPP]^2+^ with phosphotungstic acid.

**Figure 4:**
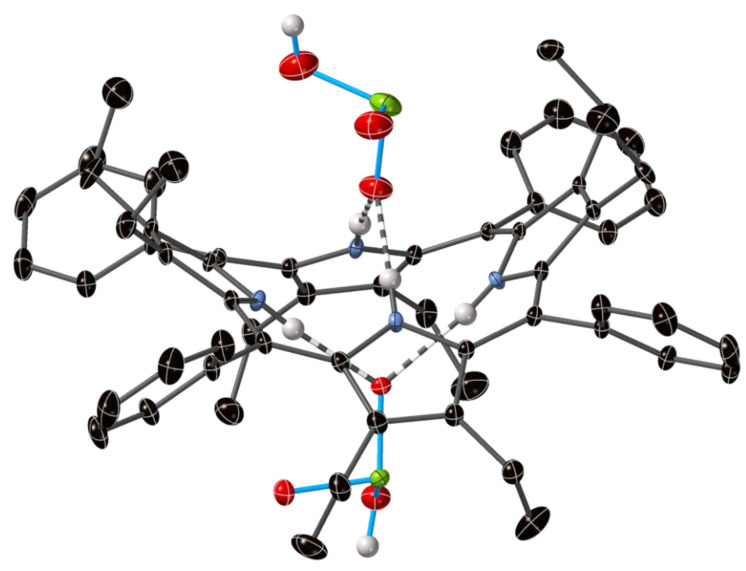
The “isolated” molecular cluster resulting from a 1:2 ratio of dication to monoanion in the structure of [H_4_**1**](HSe^IV^O_3_)_2_·(CH_3_OSe^IV^O_2_H)·3(CH_3_OH) (compound **6**); solvates and C-bound H atoms omitted for clarity.

A 1:2 ratio of dication to monoanion, as in the above relationship, forms isolated molecular clusters of balanced charge, as is the case for the hydrogen selenite monoanion; this compound [H_4_**1**](HSe^IV^O_3_)_2_·(CH_3_OSe^IV^O_2_H)·3(CH_3_OH) (compound **6**) is shown in Figure 4. A strong hydrogen bond chelate interaction is formed with an oxygen atom of each of the two monoanions. The presence of methylselenic acid as a co-precipitating agent was unintentional; while acid esterification is commonly self-catalyzed, a presence at stoichiometric level implies that porphyrin diacids may play a role in the acid catalysis under ambient conditions.

**Figure 5:**
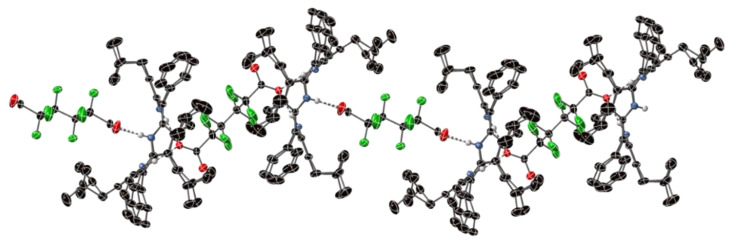
Two repeat units of the one-dimensional hydrogen-bonded chain of [H_4_OiBuTPP]^2+^ with [C_6_F_8_O_4_]^2−^ from the crystal structure of compound [H_4_**2**](O_2_C(CF_2_)_4_CO_2_)·1.5(CH_2_Cl_2_)·1.5(CH_3_OH) (compound **2**).

An example of a one-dimensional chain, that of H_4_OiBuTPP with octafluorohexanedioic acid (compound **2**), is shown in Figure 5. The expected hydrogen bond chelate activity is present, as indicated with dashed bonds; the 1:1 ratio and the interaction with both ends of the bifunctional anion is shown. The two different types of anions in the asymmetric unit interact differently with the porphyrin core, with a N–H⋯O–C–O⋯H–N pattern (O1) and a N–H⋯O⋯H–N pattern (O3). Alkyl groups project along the pseudo-*S*_4_ molecular axis, forming a pocket which encloses the counter-anion.

**Figure 6:**
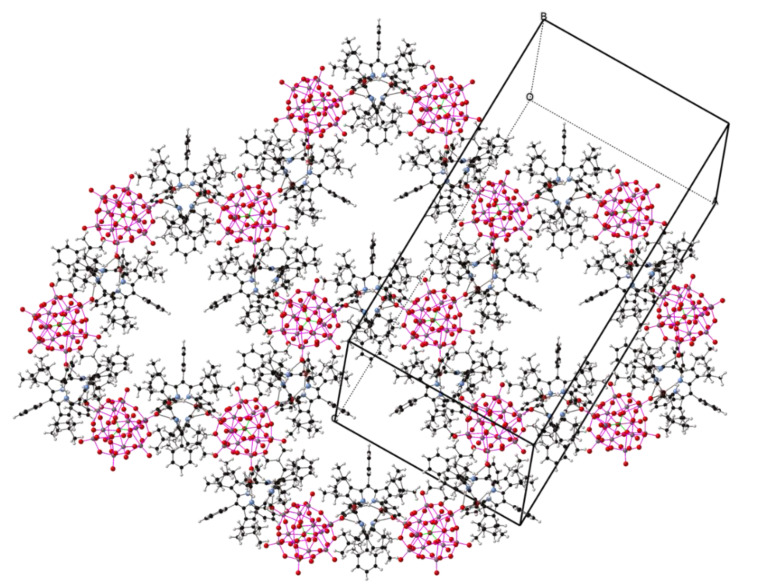
The 6,3-net structure of a single layer of the porphyrin–POM hybrid material [H_4_**2**]_3_(Mo^VI^_12_O_36_(PO_4_))_2_·60(CH_3_OH)·1.5(H_2_O). The POM components are shown in pink bonds, while atoms are represented as spheres of a fixed radius.

**Figure 7:**
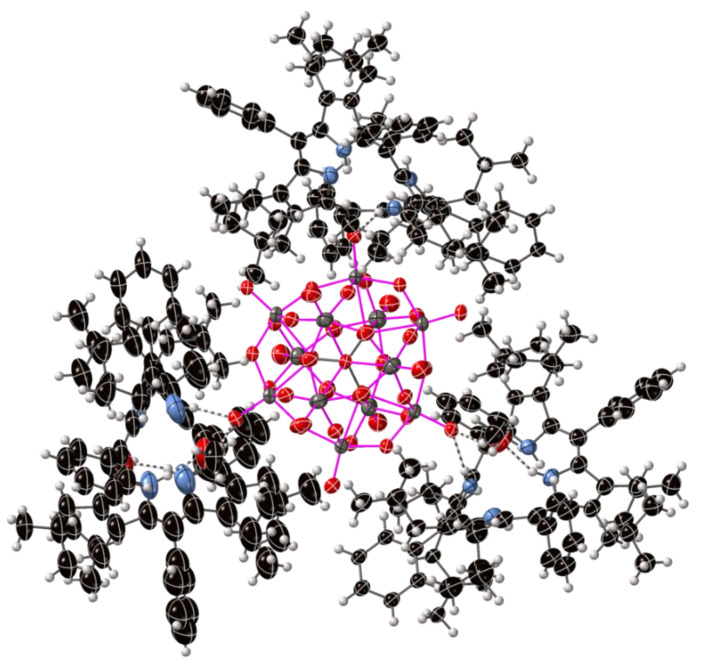
The hydrogen bonding interactions (striped bonds) around one POM node in [H_4_**2**]_3_(Mo^VI^_12_O_36_(PO_4_))_2_·60(CH_3_OH)·1.5(H_2_O) (compound **4**), in which three porphyrin diacids and adjunct solvate are arranged around the equatorial acceptor sites and a pseudo-threefold axis. The distances of H-bonding interactions are listed in Table 1.

The simplest example we have observed of a two-dimensional polymeric arrangement is the 6,3-net of OiBuTPP with the Keggin phosphomolybdate POM shown in Figure 6 (compound **4**). The crystal structure shows many interactions between the six unique porphyrin units and the four POM units via terminal O atoms, with water and methanol solvates inserting into “X”- and “Y”-type multifactor interactions, an example of which is shown in Figure S4.5. The large size of the POM counter-anion, combined with the bulk of the porphyrin and the controlled arrangement of these layers, yield a large unit cell; each porphyrin–POM sheet contains irregular hexagonal interstices of disordered solvent which account for 35% of the volume of the crystal. Some disorder could be modelled as distinct atomic positions; however, the variability in thermal ellipsoids between porphyrin components is clearly visible in Figure 7.

**Figure 8:**
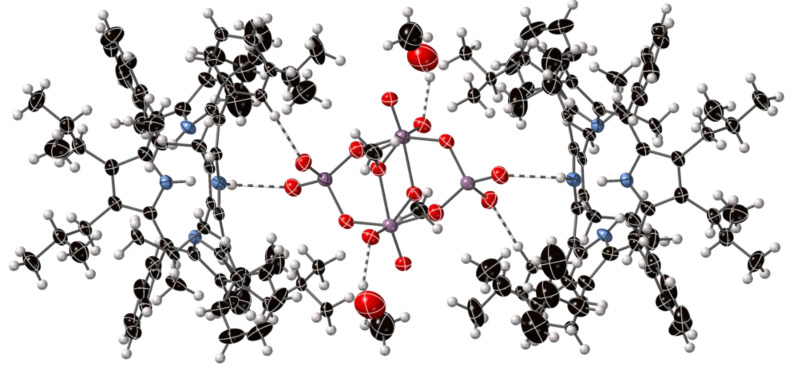
The N–H⋯O and C–H⋯O interactions of the serendipitous precipitation of the [H_3_**2**]^+^ porphyrinium monocation with an in situ-generated [Mo_4_O_12_(OMe)_2_]^2−^ POM unit (compound **9**).

Attempts to form a similar “net”, in which a POM with a 4-charge may act as a 4-connected node by analogy were unsuccessful; under the crystallization conditions, the polyoxometalate unit metamorphosed to a Mo_4_O_12_(OMe)_2_ dianion chelated by two 22*H*-porphyrinium monocations, as shown in Figure 8 (compound **9**). A beautiful example of the proposed four-connected, three-dimensional net has been reported recently by Yamazaki et. al. with SV_2_W_10_O_40_ as the counterion [36].

**Figure 9:**
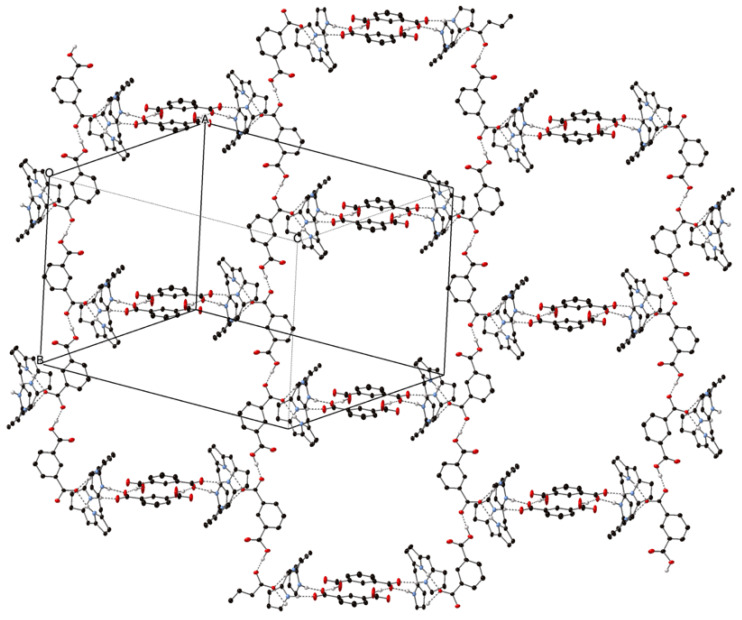
The irregular hexagonal net of the porphyrindiium bis(hydrogen phthalate) crystal of compound H_4_OiBuTPP(H(Pth))_2_. Side chains on the porphyrin component, H atoms not involved in H-bonding, and solvates are omitted.

The introduction of counter anions that are able to form symmetrical hydrogen-bonding interactions, such as the phthalate monoanion, similarly forms polymeric and interconnected “nets”. The hexagonal network H_4_OiBuTPP(H(Pth))_2_, consisting of a 2:1 ratio of the two components, is shown in Figure 9. In this compound, the sharing of protons over two chemically equivalent positions of the anion dimer results in a reduced distance between these anion pairs at 2.540(3) and 2.599(3) Å (D⋯A), termed a symmetrical hydrogen bond with some covalent characteristics [43].

**Figure 10:**
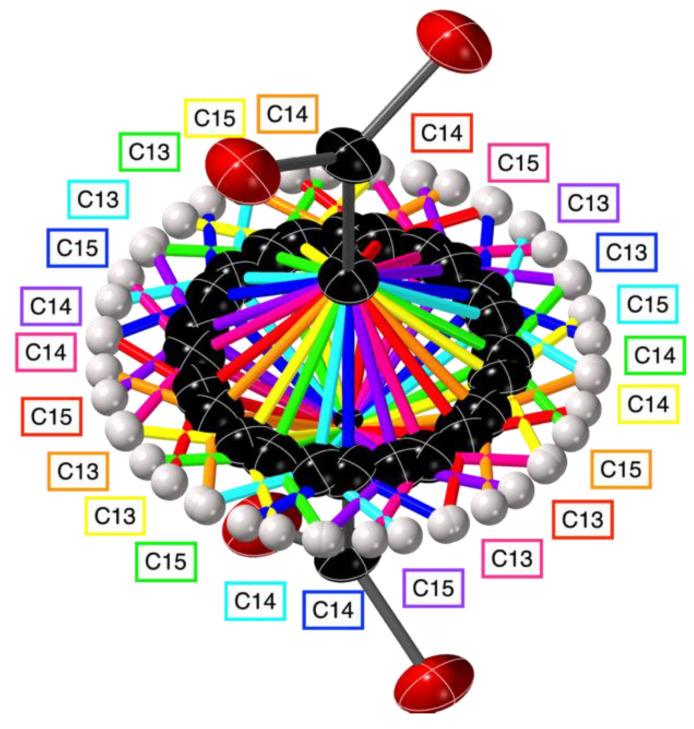
Disorder of the methylene bridges within the crystallographically identified bicyclo[1.1.1]pentane-1,3-dicarboxylate; each of the eight colors (red, orange, yellow, green, light blue, dark blue, purple, and pink) represents a 1/8 occupancy of the disordered (CH_2_)_3_ units.

**Figure 11:**
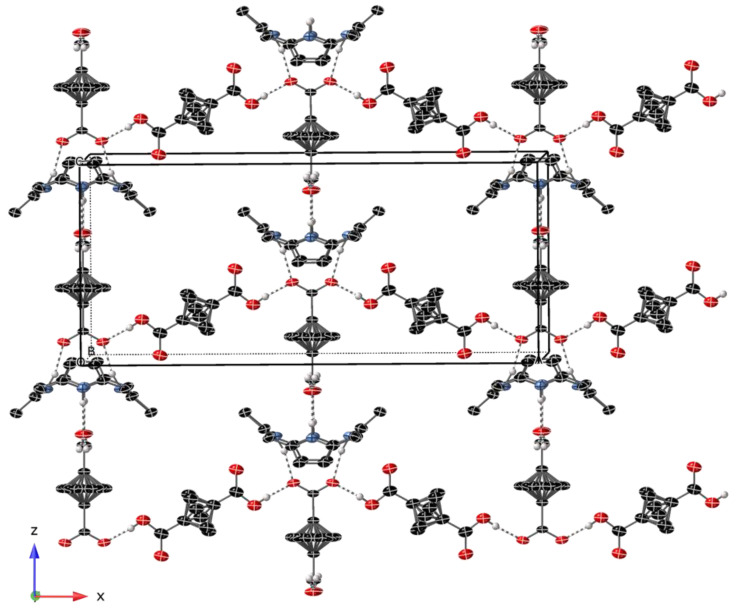
A two-dimensional ‘slice’ of the intricate three-dimensional hydrogen-bonded network of [H_4_OETPP][BCP(CO_2_)_2_]·2(BCP(CO_2_H)_2_).

A choice to include an excess of acid species was made under the assumption that, forming at interfaces, these materials were not precipitating under equilibrium conditions, and that a strict ratio of components would lead to a basic solution which would hinder crystal growth. This acid excess resulted in a densely interconnected net with the bicyclo[1.1.1]pentane-1,3-dicarboxylate counter-anion structure; the inclusion of additional protonated BCP diacid species formed a tangled hydrogen bonded network, in which each porphyrin is linked to six others. As we have previously discussed, the three-fold symmetry of the bicyclo[1.1.1]pentane unit over a high symmetry site can result in near-complete rotational disorder of the methylene bridges [44]; this is shown in Figure 10, with the simple hydrogen-bonded chain of porphyrin with bicyclo[1.1.1]pentane-1,3-dicarboxylic acid inset within the larger hydrogen-bonded network in Figure 11.

The crystal structures of porphyrindiium dication structures with acetate, malonate, decane-1,10-dicarboxylic acid, bromide, and chloride are listed in Tables S1–S3, alongside critical values derived from the crystal structures. Images of these structures are available in Figures S1.1–S10.4, with crystal information files available as Supplementary Information.

### 2.3. NSD Analysis

Each of the porphyrin macrocycles presented demonstrates a “saddle” conformation; the degree of specific macrocyclic conformation can be quantified by NSD studies [7]. As expected, the projective “saddle” conformation common to most porphyrin diacids aligns with the first and second normal modes of B_2u_ representation, with values tabulated in Table 3.

**Table 3:** The prominent out-of-plane NSD values for the structures discussed in this paper. ^†^ B_2u_(2) modes are relative to B_2u_(1) sign.

Compound Formula	Compound Number	|B_2u_(1)| (Å)	B_2u_(2) ^†^ (Å)	B_1u_(1) (Å)	A_2u_(1) (Å)
		“Saddle”		“Ruffle”	“Dome”
[H_4_**2**](HSO_4_)_2_·H_2_O·3(MeOH)	**1**	3.92	−0.68	0.44	0.04
[H_4_**2**](O_2_C(CF_2_)_4_CO_2_)·1.5(CH_2_Cl_2_)·1.5(CH_3_OH)	**2**	3.89	−0.65	−0.15	0.14
[H_4_**2**](Br)_2_·1.6(CH_3_OH)	**3**	4.08	−0.73	0.25	−0.11
[H_4_**2**]_3_(Mo^VI^_12_O_36_(PO_4_))_2_·60(CH_3_OH)·1.5(H_2_O)	**4**(**1**)	3.86	−0.64	0	−0.06
	**4**(**2**)	3.95	−0.72	−0.41	0.05
	**4**(**3**)	4.04	−0.84	−0.37	0.12
	**4**(**4**)	3.86	−0.66	0.16	−0.04
	**4**(**5**)	3.93	−0.69	0.15	0.01
	**4**(**6**)	3.98	−0.74	−0.06	0
[H_4_**1**](O_2_C(C(CH_2_)_3_C)CO_2_)·(HO_2_C(C(CH_2_)_3_C)CO_2_H)	**5**	4.07	−0.64	0	0
[H_4_**1**](HSe^IV^O_3_)_2_·(CH_3_Ose^IV^O_2_H)·3(CH_3_OH)	**6**	3.61	−0.76	−0.01	−0.05
[H_4_**1**](O_2_C(CH_2_)_10_CO_2_)·4(CH_3_OH)·H_2_O	**7**	3.94	−0.67	0.18	0.06
[H_4_**2**](O_2_C(CH_2_)CO_2_H)Cl_0.7_Br_0.3_·2(CH_3_OH)	**8**	4.08	−0.71	−0.23	−0.18
[H_3_**2**]_2_(Mo^VI^_4_O_12_(MeO)_2_)·6(CH_3_OH)	**9**	3.87	−0.72	0.11	0.05
[H_3_**2**](O_2_CCH_3_)·0.31(H_2_O) 1.25(CH_2_Cl_2_)·0.69(CH_3_OH)	**10**	4.06	−0.76	−0.27	−0.18
[H_4_**2**](O_2_CC_6_H_4_CO_2_H)_2_·2.5(CH_3_OH)·0.55(CH_2_Cl_2_)·0.6(H_2_O)	**11**	3.97	−0.75	0.02	0.07

^†^ relative to B_2u_(1) sign.

From this data, we can observe that the values are generally clustered with the previously identified diacid cluster; the commonly encountered “ruffle” and “dome” shape deviations from the accessed *D*_2*d*_ symmetry are minimal. The B_2u_ values are clustered around B_2u_(1) with 3.94, and B_2u_(2) with −0.71, conformationally in line with previous examples of H_4_OETPP^2+^ compounds [45].

## 3. Discussion

The near-identical morphology and interaction profile of the O(alkyl)TPP examples with various anions imply that this unit can be incorporated into designed systems readily. A two-component interaction type—rarely observed previously but common here—coerces reaction partners to close contact. As such, the potential applications to catalysis are readily apparent. This result is congruent with our previously proposed interaction profile for the related bifunctional catalyst type, with the free base Et*_x_*TPP series (*x* = 2, 4, 6, 8), demonstrating acceleration of the sulfa-Michael reaction on promotion of sufficient nonplanarity [46].

Dodecasubstituted porphyrins can act in a similar manner to the ‘pocket’ porphyrins [47], in which *ortho*- and *meta*-substituents of meso-aryl units act as steric blockers to interaction with the porphyrin core. In dodecasubstituted examples, alkyl groups on b-positions are projected perpendicular to the porphyrin plane and can potentially act as similar blockers, enforcing regulation of intermolecular interactions. This can be seen clearly with the peripheral iBu groups (in comparison to the ethyl groups of OETPP) with increased bulk, encroaching further towards the porphyrin central axis. The use of peripheral substitution as a biomimetic pseudo-allostery in chemical systems is potentially a rich area, combining chemical stability with biological selectivity.

The close-contact interactions of the nonplanar H_4-_porphyrin diacid with POM has been assumed in previous studies, and we can show how that polymeric interaction is able to manifest in terms of supramolecular dimensionality. Previous studies have indicated a 3:2 ratio of porphyrin/POM for the “3-”Keggin POM [SW_11_VO_40_]^3−^ with H_4_TPP^2+^ (Figure 1, R^1^ = Ph, R^2^ = H) [48]. Reasonable assertions can be made that the supramolecular packing and interaction profile in that case would resemble our two-dimensional hexagonal net of [H_4_**2**]_3_(PMo_12_O_40_)_2_ but with the “I” interaction profile (see Figure 2) and unknown interlayer packing.

Taking as our example the simplest polymeric supramolecular design element, that of a one-dimensional chain, we can assert that the entire range of ordered two- and three-dimensional materials should be accessible under self-organization. The combination of a polyfunctional carboxylic acid, such as those engaged in MOF synthesis (e.g., 1,3,5-tris(4-carboxyphenyl)benzene) with a nonplanar porphyrin, is an easy method of generating a defined spacing, or dispersion, of these active centers while controlling dimensionality.

The choice of protic solvents in the synthesis of oxoanion materials results in the inclusion of these as solvates adjacent to the porphyrin core. This demonstrates elegantly that when distortion from planarity is enhanced, the displacement of the anion by an analyte is not strictly necessary for interaction with the porphyrin core and, thus, offers a handle for increasing chemoselectivity.

The coordination of polyaxiality here, given the narrow range of interaction profiles, is indicative of how free base or protonated nonplanar porphyrins may act on the surface of metal oxides. Effective measurements on porphyrin anchoring forming ordered and face-selective aligned materials is only beginning to be enabled by microscopy [49]. Binding to terminal oxo- or related species (e.g., layered double hydroxides [50]) through the core allows for designed single-molecule heterojunctions with favorable binding. This combination of a pathway for electron transfer (from, e.g., TiO_2_ [51]) and proton availability enables H atom exchange and a rich catalytic potential.

## 4. Materials and Methods

### 4.1. Synthesis

Here, 2,3,7,8,12,13,17,18-Octaethyl-5,10,15,20-tetraphenylporphyrin (OETPP, Figure 1, R^1^ = Ph, R^2^ = Et) and 2,3,7,8,12,13,17,18-octakis(2-methylpropyl)-5,10,15,20-tetraphenylporphyrin (OiBuTPP, Figure 1, R^1^ = Ph, R^2^ = 2-MePr) were synthesized by previously published procedures [52,53]. All other chemicals were ordered from commercial suppliers and used as received.

Porphyrin free bases were dissolved in dichloromethane and layered under a solution of the acid species in methanol, with a buffer layer of 1:1 MeOH/DCM, inside of a partially covered crystallization tube [54]. Nucleation occurred at the interface of these two solutions upon slow evaporation-induced concentration of the supernatant. Crystals suitable for X-ray diffraction formed upon standing for three weeks at room temperature. No special care was taken to exclude water from hydrated reactants; as such, some of these crystal structures appear as hydrates. Acetate was formed in situ from the decomposition of the bicyclo[1.1.1]pentane-1,3-dicarboxylic acid.

### 4.2. Crystal Structure Analysis

Diffraction patterns were obtained using MoKα or CuKα radiation on a Bruker D8 Quest ADVANCE-ECO or APEX-II Duo device as indicated in Tables S1–S3 (Supplementary Materials Pages 2–4), and data were reduced by standard procedures using the Bruker suite. Structure solution was provided with ShelXT [55] and refinement of the structure was performed with ShelXL [56] in Shelxle [57]. Disordered solvates within interstices were accounted for with the SQUEEZE routine in PLATON as indicated [58]. The NSD analyses were performed using the online tools available at https://www.sengegroup.eu/nsd (accessed on 1 October 2022) [7].

The isobutyl groups in these crystal structures often display two orientations, as has been noted previously for, e.g., isopropyl groups on highly distorted porphyrins. Individual structure restraints are listed in the CIF files; generally, H atoms were refined as riding thermal parameter spheres, and N–H and O–H distances were attempted to be refined freely, and only constrained when necessary. All non-hydrogen atoms were refined with anisotropic thermal ellipsoid parameters; individual components of disorder were identified by looking for bimodality in the electron density map and refined freely to a sensible sum occupancy. Disordered components were held to SIMU restraints for accurate occupancy.

## 5. Conclusions

Nonplanar diacid porphyrin clusters—a potentially rich class of photonic materials—are expanded to include a variety of metal-bearing anion counterparts for the self-assembly of low-dimensional components. The dimensionality of these materials can be controlled by a simple charge-balance rule for the formation of one- and two-dimensional materials. The potential number of dyad combinations is huge; nevertheless, the structural variability is low within the porphyrin component, and the expected hydrogen bond chelate is shown to be controllable by solvent inclusion and imposed nonplanarity.

The self-assembly of ionic partners to a well-ordered 2D-in-3D crystalline material suggests that these structures can be replicated on-surface in 2D, and this avenue is being explored. Given the proximity of photonic reaction center, metallocluster, and a convenient reaction ‘pocket’ in the hexagonal porphyrin–POM net, such compounds could lead the way into new, photoactive ultrathin devices. Developing general principles for the relationship between individual molecular shape and intermolecular aggregation is a materials science frontier where significant progress is being achieved [59] and, given the shape-control mechanisms available, we see a promising future for porphyrin-bearing self-assembled materials.

## Figures and Tables

**Table 1:** The compounds discussed in this paper.

Compound Number	Compound	Porphyrin	Anion	Ratio P:A
**1**	[H_4_**2**](HSO_4_)_2_·H_2_O·3(MeOH)	H_4_OiBuTPP	HSO_4_	1:2
**2**	[H_4_**2**]O_2_C(CF_2_)_4_CO_2_·1.5(CH_2_Cl_2_)·1.5(CH_3_OH)	H_4_OiBuTPP	Octafluorohexane-1,6-dicarboxylate	1:1
**3**	[H_4_**2**]Br_2_·1.6(CH_3_OH)	H_4_OiBuTPP	Br	1:2
**4**	[H_4_**2**]_3_(Mo^VI^_12_O_36_PO_4_)_2_·60(CH_3_OH)·1.5(H_2_O)	H_4_OiBuTPP	Mo_12_O_36_(PO_4_)	3:2
**5**	[H_4_**1**]O_2_C(C(CH_2_)_3_C)CO_2_·HO_2_C(C(CH_2_)_3_C)CO_2_H	H_4_OETPP	Bicyclo[1.1.1]pentane-1,3-dicarboxylate	1:1 §
**6**	[H_4_**1**](HSe^IV^O_3_)_2_·CH_3_OSe^IV^O_2_H·3(CH_3_OH)	H_4_OETPP	HSeO_3_	1:2
**7**	[H_4_**1**]O_2_C(CH_2_)_10_CO_2_·4(CH_3_OH)·H_2_O	H_4_OETPP	Dodecane-1,12-dicarboxylate	1:1
**8**	[H_4_**2**](O_2_C(CH_2_)CO_2_H)Cl_0.7_Br_0.3_·2(CH_3_OH)	H_4_OiBuTPP	Malonate, (0.3Br/0.7Cl)	1:1:1
**9**	[H_3_**2**]_2_Mo^VI^_4_O_12_(MeO)_2_·6(CH_3_OH)	H_3_OiBuTPP	Mo^VI^_4_O_12_(MeO)_2_	2:1
**10**	[H_3_**2**]O_2_CCH_3_·0.31(H_2_O) 1.25(CH_2_Cl_2_)·0.69(CH_3_OH)	H_3_OiBuTPP	Acetate	1:1
**11**	[H_4_**2**](O_2_CC_6_H_4_CO_2_H)_2_·2.5(CH_3_OH)·0.55(CH_2_Cl_2_) 0.6(H_2_O)	H_4_OiBuTPP	Hydrogen phthalate	1:2

§ Additional bicyclo[1.1.1]pentane-1,3-dicarboxylic acid is present as a co-crystallizing agent.

## Data Availability

Crystallographic Information Files (CIFs): CCDC 2202905-2202915 contain the supplementary crystallographic data for this paper. These data can be obtained free of charge via www.ccdc.cam.ac.uk/data_request/cif, or by emailing data_request@ccdc.cam.ac.uk, or by contacting The Cambridge Crystallographic Data Centre, 12 Union Road, Cambridge CB2 1EZ, UK; fax: +44-1223-336-033.

## Supplementary Materials

The following supporting information can be downloaded at: https://www.mdpi.com/article/10.3390/molecules27207060/s1, Supplementary crystallographic images and crystallographic tables.
