# Supplementary material for: Self-Organization of Porphyrin–POM Dyads: Nonplanar Diacids and Oxoanions in Low-Dimensional H-Bonding Networks
Source: Molecules. 2022 Oct 19;27(20):7060. doi: 10.3390/molecules27207060 (PMC9609041; doi:10.3390/molecules27207060)
Supplement: Supplementary file 1 [file molecules-27-07060-s001.zip › molecules-1978285-supplementary.pdf]

Supplementary Materials

Self-Organization of Porphyrin–POM Dyads: Nonplanar Diacids and Oxoanions in Low-Dimensional H-Bonding Networks

Christopher J. Kingsbury, Marc Kielmann, Brendan Twamley and Mathias O. Senge *

Crystal Structure Tables, Pages 2–4

Crystal Structure Images, Pages 5–37

Table S1. Crystallographic data for compounds **1–4.**

Compound Number	1	2	3	4
Chemical Name	22*H*,24*H*-2,3,7,8,12,13,17,18-octa(2-methylpropyl)-5,10,15,20-tetraphenylporphyrindiium bis(hydrogen sulfate) trimethanol water solvate	22*H*,24*H*-2,3,7,8,12,13,17,18-octa(2-methylpropyl)-5,10,15,20-tetraphenylporphyrindiium perfluorohexan-1,6-dioate 1.5 methanol 1.5 dichloromethane solvate	22*H*,24*H*-2,3,7,8,12,13,17,18-octa(2-methylpropyl)-5,10,15,20-tetraphenylporphyrindiium dibromide 1.6 methanol solvate	tris(22*H*,24*H*-2,3,7,8,12,13,17,18-octa(2-methylpropyl)-5,10,15,20-tetraphenylporphyrindiium) bis(phosphotungstate) 60 (methanol) 1.5 (water) solvate
Chemical Formula	$C_{79}H_{112}N_4O_{12}S_2$	$C_{85}H_{105}Cl_3F_8N_4O_{5.50}$	$C_{77.60}H_{102.40}Br_2N_4O_{1.60}$	$C_{180}H_{15}N_{24}O_{240}P_6W_{72}$
CCDC deposition #	2202906	2202907	2202908	2202915
Internal Code	TCD1244	TCD1257	TCD1258	TCD1259
Crystal System	monoclinic	triclinic	monoclinic	triclinic
Space Group	$P\ 2_1/n$	P -1	C 1 2/c 1	*P* -1
Crystal Description	needle	parallelepiped	block	plate
Crystal Color	black	green	green	Black
a (Å)	15.1405(8)	14.4393(5)	13.8262(6)	27.3256(9)
b (Å)	25.2825(14)	15.5036(5)	23.7473(11)	29.5702(10)
c (Å)	20.3511(11)	21.5705(7)	22.7591(13)	48.3874(16)
α (°)	90	94.8490(10)	90	107.2256(10)
β (°)	104.1555(19)	108.9490(10)	93.3238(15)	91.9161(10)
γ (°)	90	110.9900(10)	90	96.2705(11)
Volume ($Å^3$)	7553.6(7)	4152.3(2)	7460.0(6)	37032(2)
Crystal Size (mm^3)	0.050 × 0.140 × 0.500	0.634 × 0.11 × 0.101	0.423 × 0.181 × 0.141	0.020 × 0.175 × 0.323
Density (a.u.)	1.208	1.223	1.137	3.547
μ (mm^{-1})	0.133	0.180	1.131	22.381
Absorption Corr.	multi-scan	multi-scan	multi-scan	multi-scan
Tmin, Tmax	0.6099	0.6967, 0.7456	0.6886, 0.7453	0.225, 0.861
Temp (K)	100(2)	200(2)	200(2)	100(2)
λ (Å)	0.71073	0.71073	0.71073	0.71073
Radiation type	MoKα	MoKα	MoKα	MoKα
Measured Reflections	73934	62914	61326	670385
$θ_{max}$	25.526	27.576	26.155	26.172
Scan Mode	ω & φ	ω & φ	ω & φ	ω & φ
R_1 (all)	0.2202	0.1147	0.0696	0.2268
R_1 (I > 2σ)	0.0930	0.0666	0.0486	0.0920
wR_2 (all)	0.2701	0.2115	0.1571	0.3161
wR_2 (I > 2σ)	0.2043	0.1797	0.1411	0.2246
Goodness of Fit	1.014	1.028	1.020	0.969
H- atoms ref.	mixed	mixed	mixed	mixed
Independent Refl.	14030	19115	7420	147049
Indep. Refl. (I > 2σ)	6146	11773	5624	59181
Max and Min residual	0.776, -0.480	0.930, -0.626	0.561, -0.670	5.056, -2.098
Data / Parameters / Restraints	14030/ 1025/ 300	19115 / 1180 / 518	7420 / 591 / 565	147049/ 6693/ 9237

Table S2. Crystallographic data for compounds **5–8**.

Compound Number	5	6	7	8
Chemical Name	[22H,24H]-2,3,7,8,12,13,17,18-octaethyl-5,10,15,20-tetraphenylporphyrindi-ium bicyclo[1.1.1]pentane-1,3-dicarboxylate bis(bicyclo[1.1.1]pentane-1,3-dicarboxylic acid)	[22H,24H]-2,3,7,8,12,13,17,18-octaethyl-5,10,15,20-tetraphenylporphyrindi-ium bis(hydrogenselenite) (hydrogen methylselenite) tris(methanol solvate)	[[22H,24H]-2,3,7,8,12,13,17,18-octaethyl-5,10,15,20-tetraphenylporphyrindi-ium dodecane-1,12-dicarboxylate aqua tetrakis(methanol) solvate	[22H,24H]-2,3,7,8,12,13,17,18-octaethyl-5,10,15,20-tetraphenylporphyrindi-iumhydrogen malonate 0.7(chloride) 0.3(bromide) bis(methanol solvate)
Chemical Formula	$C_{81}H_{82}N_4O_{12}$	$C_{64}H_{82}N_4O_{12}Se_3$	$C_{76}H_{102}N_4O_9$	$C_{81}H_{107}Br_{0.30}Cl_{0.70}N_4O_6$
CCDC deposition #	2202905	2202911	2202910	2202913
Internal Code	TCD1278	TCD1291	TCD1293	TCD1304
Crystal System	tetragonal	triclinic	triclinic	monoclinic
Space Group	*I* 4₁/*amd*	*P* -1	P -1	*P* 2₁/*c*
Crystal Description	needle	fragment	rod	block
Crystal Color	clear green	blue	black	blue
a (Å)	25.600(18)	12.8842(5)	12.4011(5)	13.8046(6)
b (Å)	25.600(18)	13.7488(5)	13.5687(6)	23.2206(10)
c (Å)	11.218(8)	19.9988(7)	23.1278(10)	22.6782(9)
α (°)	90	109.1457(11)	78.691(2)	90
β (°)	90	93.5159(13)	88.173(2)	93.951(2)
γ (°)	90	107.9313(11)	63.693(2)	90
Volume ($Å^3$)	7352(11)	3131.8(2)	3414.1(3)	7252.2(5)
Crystal Size (mm^3)	0.310 × 0.040 × 0.040	0.182 × 0.209 × 0.422	0.426 × 0.104 × 0.059	0.050 × 0.110 × 0.350
Density (a.u.)	1.181	1.417	1.182	1.174
μ (mm^{-1})	0.635	1.826	0.077	0.980
Absorption Correction	multi-scan	multi-scan	multi-scan	multi-scan
Tmin, Tmax	0.5090 , 0.7515	0.6212, 0.7461	0.6330 , 0.7454	0.6230, 0.7531
Temp (K)	100(2)	100(2)	100(2)	100(2)
λ (Å)	1.54178	0.71073	0.71073	1.54178
Radiation type	CuKα	MoKα	MoKα	CuKα
Measured Reflections	15736	84984	45792	60932
$θ_{max}$	59.094	30.664	26.504	68.458
Scan Mode	ω & φ	ω & φ	ω & φ	ω & φ
R_1 (all)	0.1329	0.0857	0.1767	0.0683
R_1 (I > 2σ)	0.0791	0.0607	0.0832	0.0567
wR_2 (all)	0.2492	0.1660	0.2807	0.1640
wR_2 (I > 2σ)	0.2090	0.1518	0.2206	0.1545
Goodness of Fit	1.051	1.043	1.049	1.019
H- atoms ref.	mixed	mixed	mixed	mixed
Independent Refl.	1396	19104	14032	13311
Ind. Refl. (I > 2σ)	831	14446	7000	10829
Max and Min residual	0.265, -0.380	2.399, -3.810	0.785, -0.949	1.085, -0.252
Data / Parameters / Restraints	1396 / 190 / 233	19104/ 779/ 249	14032 / 1014 / 600	13311/ 909/ 335

Table S3. Crystallographic data for compounds **9–11**.

Compound Number	9	10	11
Chemical Name	bis([22H]-2,3,7,8,12,13,17,18-octa(2-methylpropyl)-5,10,15,20-tetraphenylporphyrinium) bis(μ2-methoxy)tetramolybdate hexakis(methanol solvate)	[22H]-2,3,7,8,12,13,17,18-octa(2-methylpropyl)-5,10,15-20-tetraphenylporphyrinium acetate 0.308(methanol) 0.692(water) 1.25 dichloromethane solvate	[22H,24H]-2,3,7,8,12,13,17,18-octaethyl-5,10,15,20-tetraphenylporphyrindi-ium bis(monohydrogen phthalate) 3.1(methanol) 0.55(DCM) solvate.
Chemical Formula	$C_{160}H_{220}Mo_4N_8O_{20}$	$C_{79.56}H_{103.12}Cl_{2.50}N_4O_3$	$C_{79.65}H_{87.50}Cl_{1.10}N_4O_{11.10}$
CCDC deposition #	2202909	2202912	2202914
Internal Code	TCD1305b	TCD1310	TCD1330
Crystal System	triclinic	monoclinic	monoclinic
Space Group	*P* -1	*C* 2/*c*	*C* 2/*c*
Crystal Description	fragment	block	fragment
Crystal Colour	blue	black	green
a (Å)	13.8000(6)	13.8213(7)	25.5743(12)
b (Å)	13.8299(6)	23.0945(12)	18.7396(9)
c (Å)	20.9701(10)	22.7312(12)	30.1265(14)
α (°)	85.168(2)	90	90
β (°)	80.484(2)	94.524(2)	91.6840(10)
γ (°)	78.583(2)	90	90
Volume ($Å^3$)	3863.5(3)	7233.1(6)	14432.0(12)
Crystal Size (mm^3)	0.055 × 0.101 × 0.210	0.122 × 0.310 × 0.344	0.34 × 0.18 × 0.1
Density (a.u.)	1.272	1.150	1.213
μ (mm^{-1})	0.383	0.158	0.119
Absorption Correction	multi-scan	multi-scan	multi-scan
Tmin, Tmax	0.7043, 0.7454	0.6909, 0.7454	0.7009, 0.7457
Temp (K)	100(2)	100(2)	100(2)
λ (Å)	0.71073	0.71073	0.71073
Radiation type	MoKα	MoKα	MoKα
Measured Reflections	61475	25986	98719
θ_{max}	25.750	26.925	26.500
Scan Mode	ω & φ	ω & φ	ω & φ
R_1 (all)	0.1556	0.0831	0.1243
R_1 ($I > 2\sigma$)	0.0743	0.0613	0.0788
wR_2 (all)	0.2104	0.1803	0.2631
wR_2 ($I > 2\sigma$)	0.1804	0.1610	0.2247
Goodness of Fit	1.011	1.030	1.037
H- atoms ref.	mixed	mixed	mixed
Independent Refl.	14761	7781	14952
Ind. Refl. ($I > 2\sigma$)	8035	5898	9135
Max and Min residual	0.786, -0.848	0.524, -0.446	1.347, -1.446
Data / Parameters / Restraints	14761/ 1041/ 715	7781/ 607/ 464	14952 / 1033 / 383

Supplementary crystal structure images

S1; Supplementary images for Compound 1

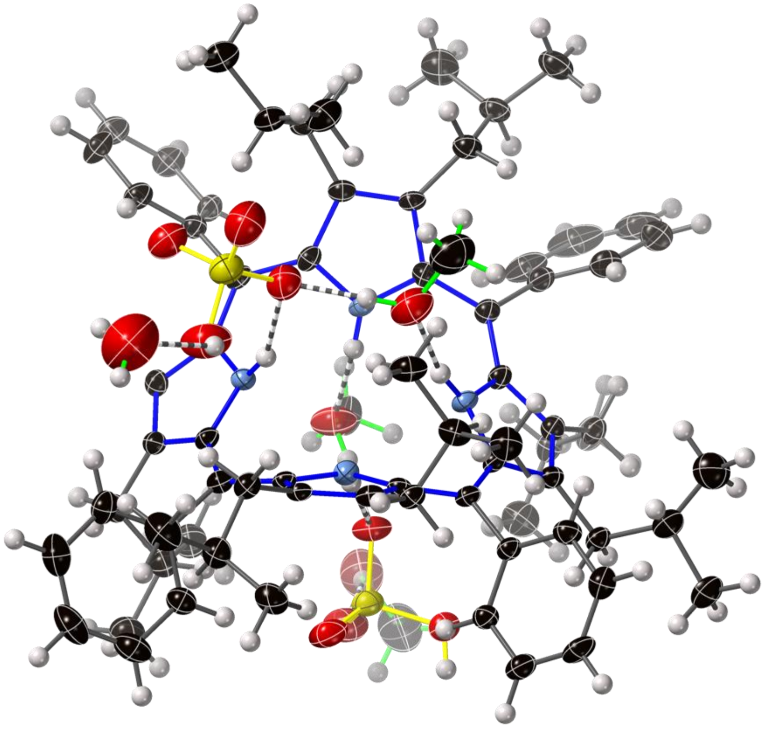

Figure S1.1; The porphyrin interactions of $[H_4OiBuTPP][HSO_4]_2(MeOH)_3(H_2O)$; the porphyrin core is shown with blue bonds, bisulfate anions in yellow, and solvates in green. Ellipsoids are shown at 50%, H atoms as spheres of fixed radius.

S2; Supplementary images for Compound 2

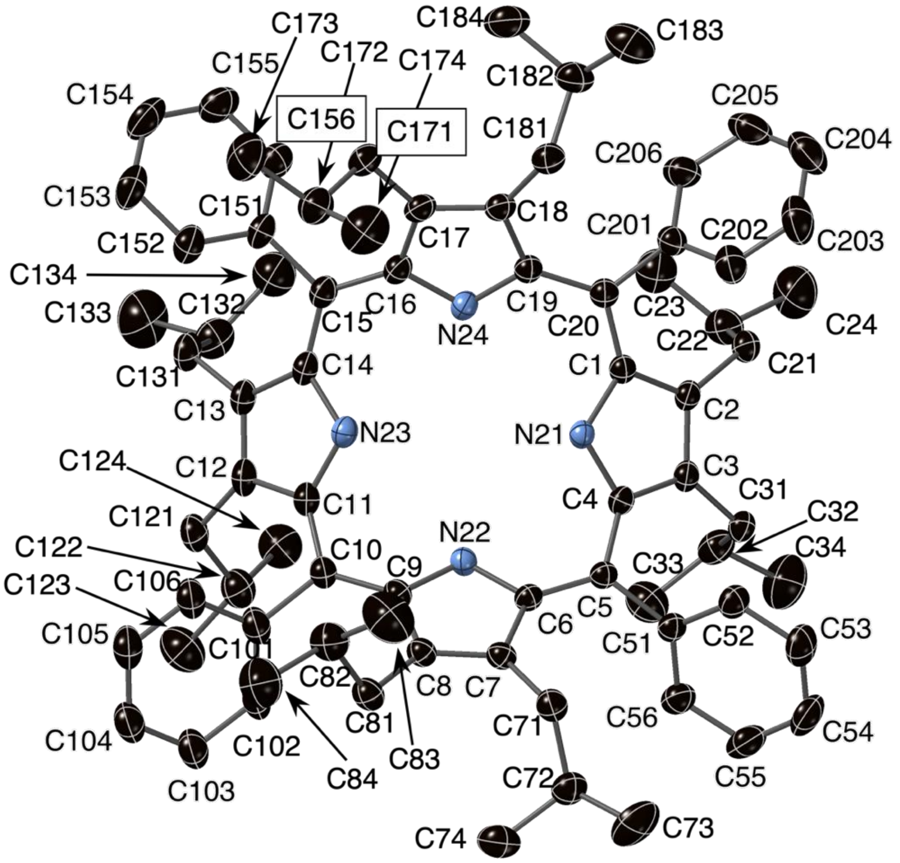

Figure S2.1; labelled diagram of the porphyrin in the crystal structure of $[H_4OiBuTPP](O_2C(CF_2)_4CO_2)_2(CH_2Cl_2)_{1.5}(MeOH)_{1.5}$. Ellipsoids at 50%, H-atoms omitted.

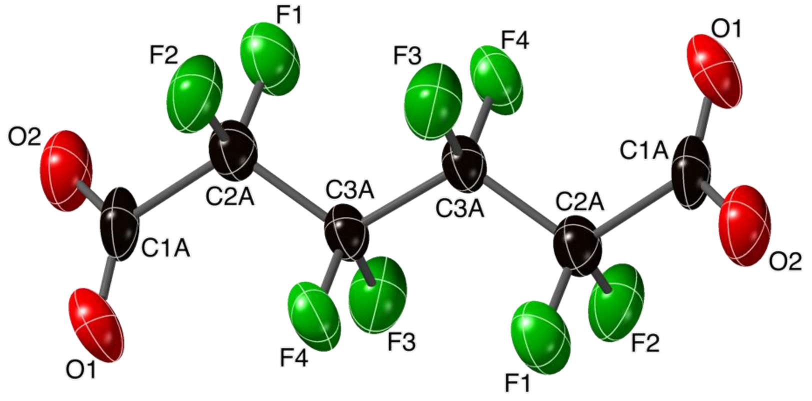

(a)

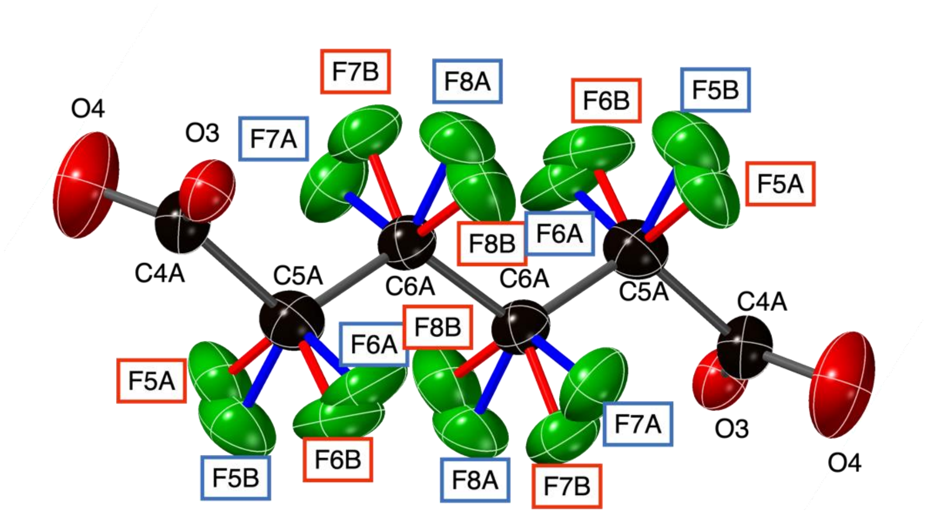

(b)

Figure S2.2(a,b) the two counteranions in the structure of $[H_4OiBuTPP](O_2C(CF_2)_4CO_2)_2(CH_2Cl_2)_{1.5}(MeOH)_{1.5}$; (b) disorder of the fluoride units across two orientations (A (0.56) and B (0.44)) is shown.

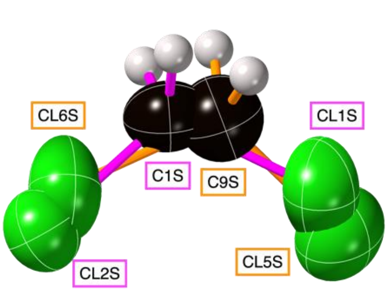

(a)

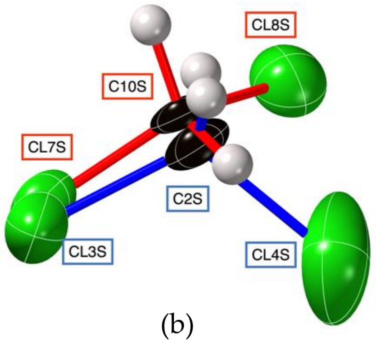

(b)

Figure S2.3(a,b) Disorder of the dichloromethane solvate;

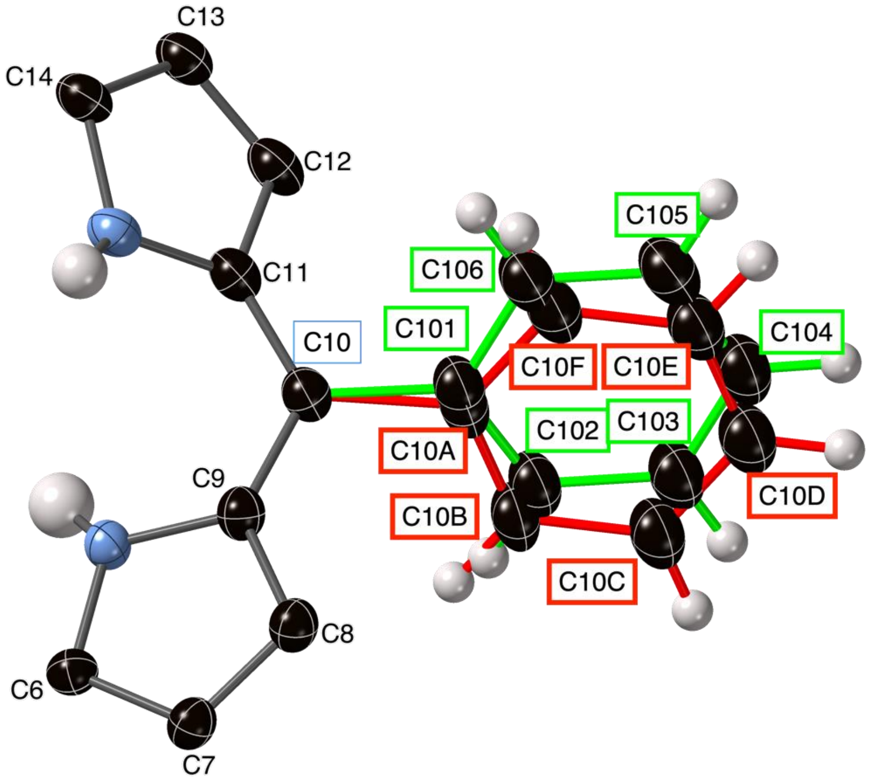

Figure S2.4; Disorder of the C10-appended ring, fixed to occupancy of 0.7 (101-106) and 0.3 (10A-10F)

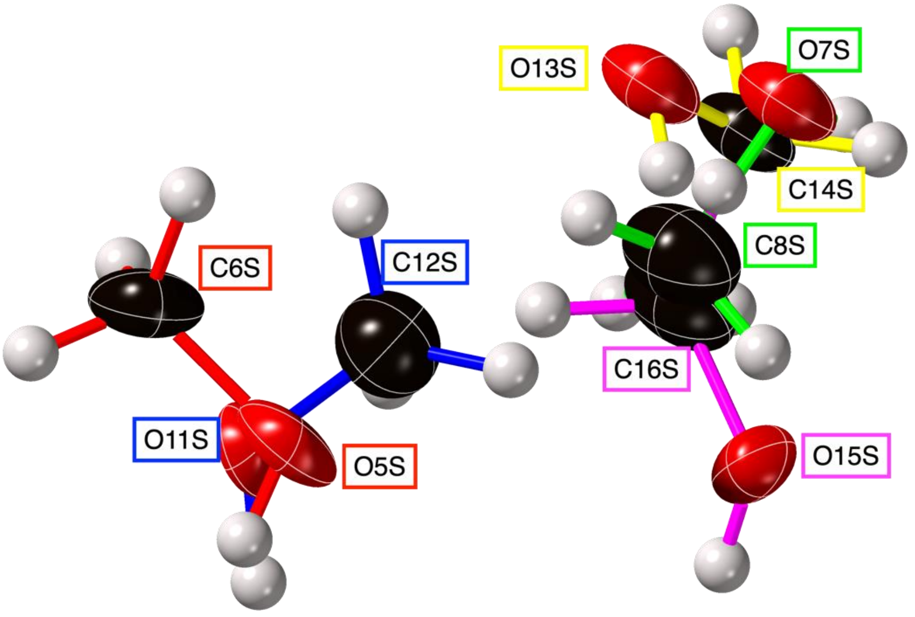

Figure S2.5; Disorder of the methanol components at occupancy red:0.4, blue:0.1, purple:0.3, green:0.5, yellow:0.2.

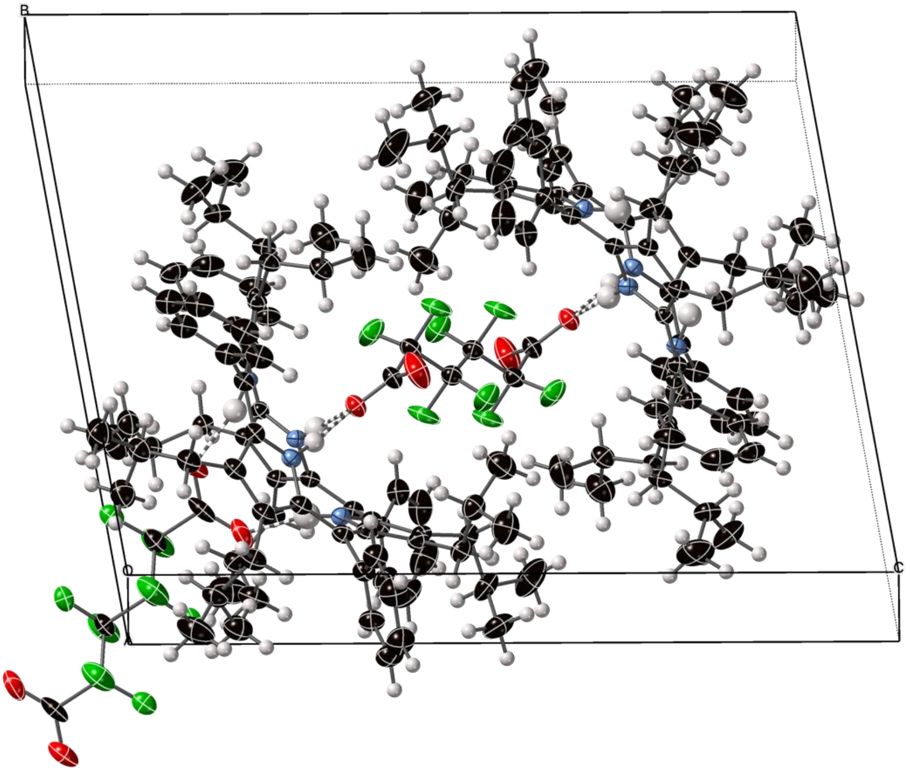

Figure S2.6; the chain structure in the unit cell of $[H_4OiBuTPP](O_2C(CF_2)_4CO_2)_2(CH_2Cl_2)_{1.5}(MeOH)_{1.5}$; solvates are omitted.

S3; Supplementary images for Compound 3

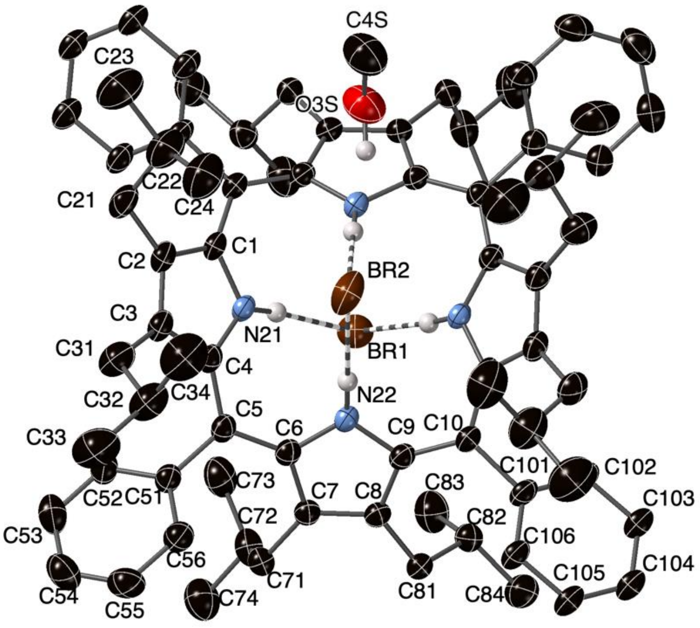

Figure S3.1; The porphyrin unit in [H_4OiBuTPP]$(Br)_2(MeOH)_{1.6}$ with the asymmetric unit labelled. Unlabelled atoms are related to labelled atoms by a 2-fold axis coincident with the Br···Br axis.

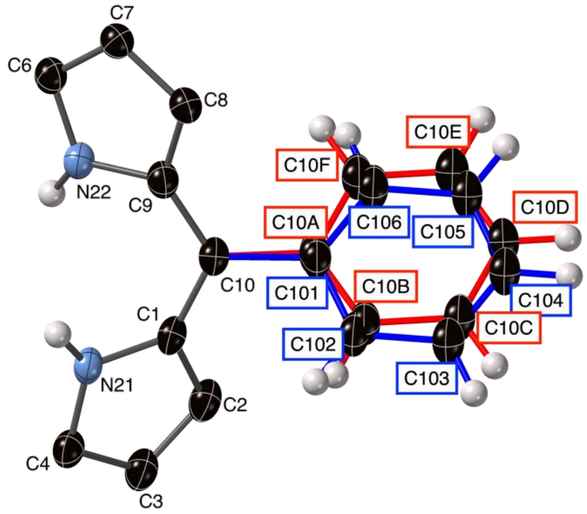

Figure S3.2; disorder in the C10-appended ring; 54% C101-106 : 46% C10A-10F

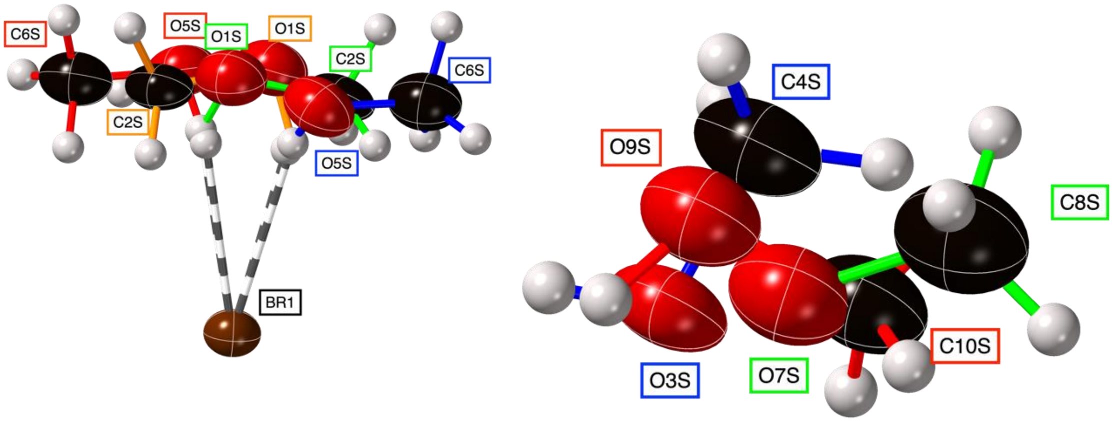

Figure S3.3; disorder in the methanol solvates (a) 15% each (red,orange,green,blue) (b) 0.2 each green, blue, 0.1 red.

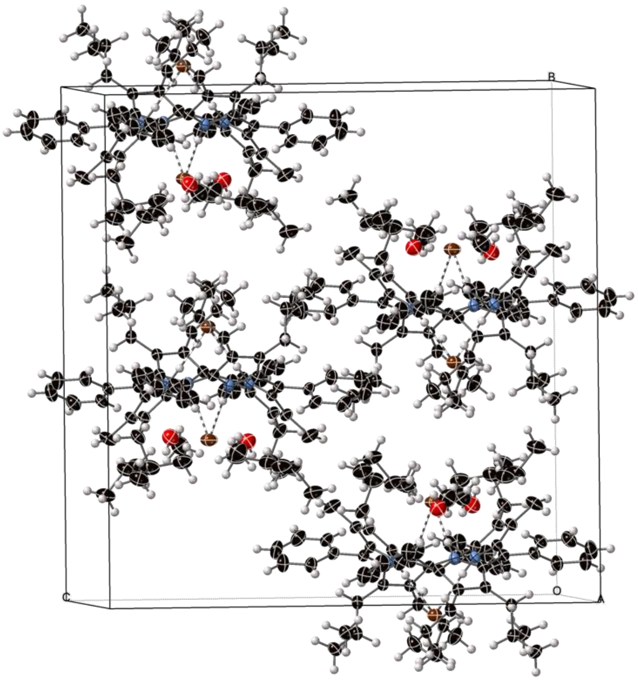

Figure S3.4; molecules within the unit cell of $[H_4OiBuTPP](Br)_2(MeOH)_{1.6}$

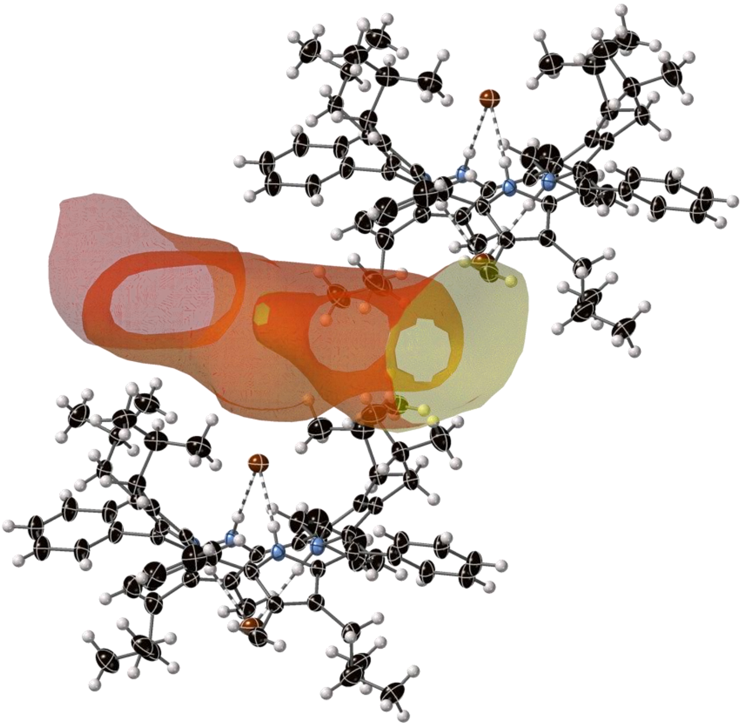

Figure S3.5; Solvent channel within the structure (1.4 Å probe)

S4; Supplementary images for Compound 4

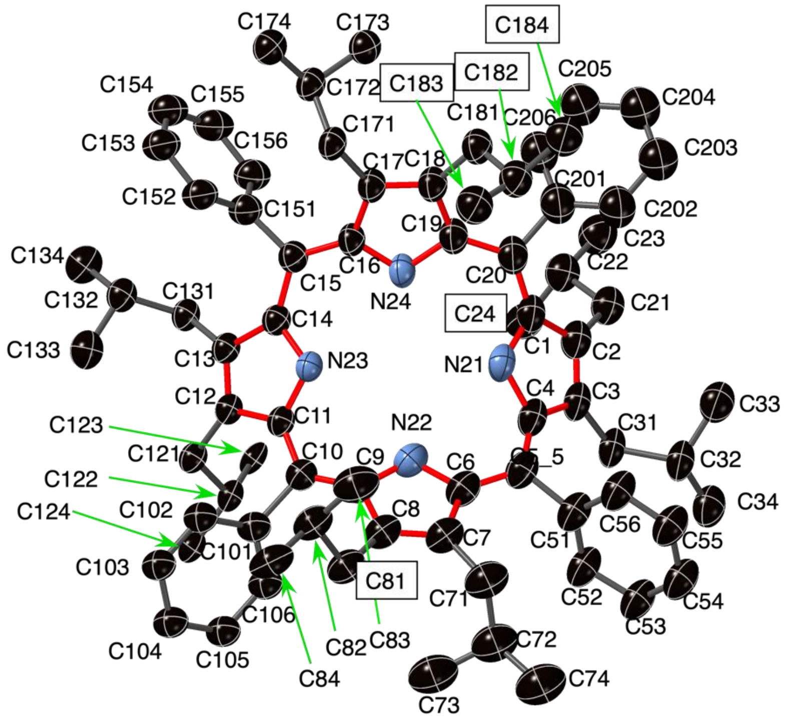

Figure S4.1; Labelled diagram of the porphyrin core in $[H_4OiBuTPP]_3(Mo_{12}O_{40})_2(MeOH)_{55}(H_2O)_{1.5}$; six equivalent porphyrin molecules were found in the unit cell, all sharing the same connectivity and numbering system.

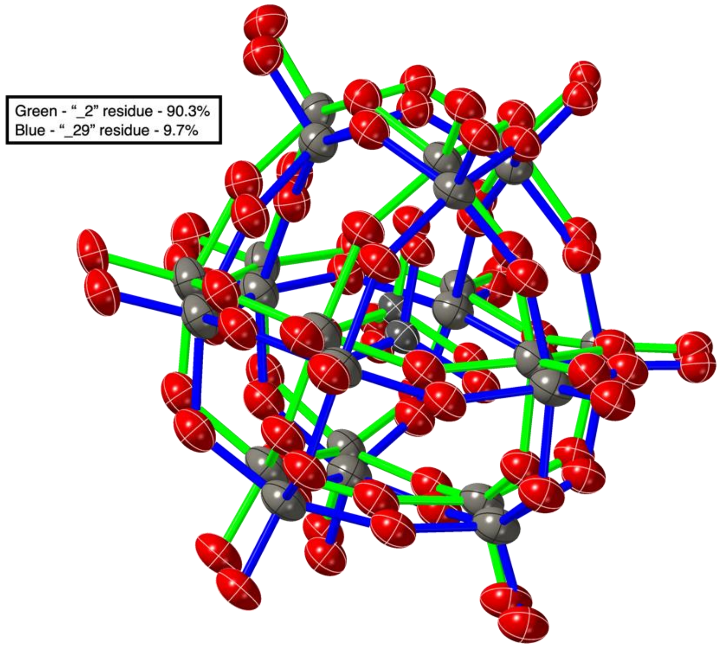

Figure S4.2; Disorder of one of the $Mo_{12}O_{40}$ clusters across two positions.

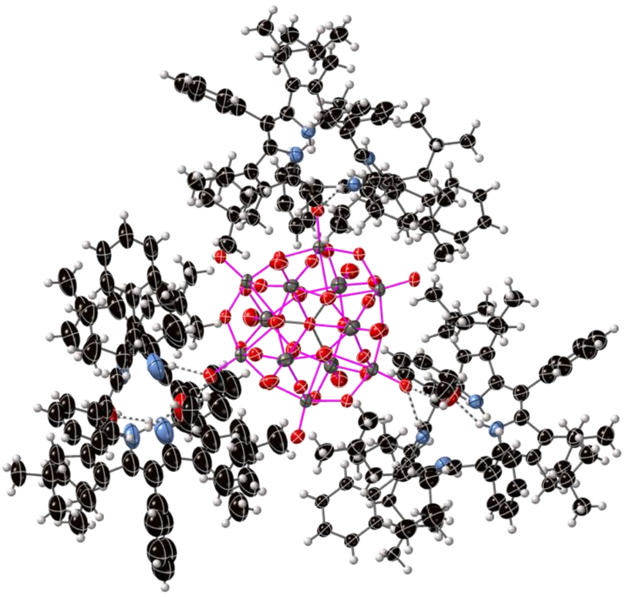

Figure S4.3; three porphyrin compounds associated with a 'node' $Mo_{12}O_{40}$ trianion.

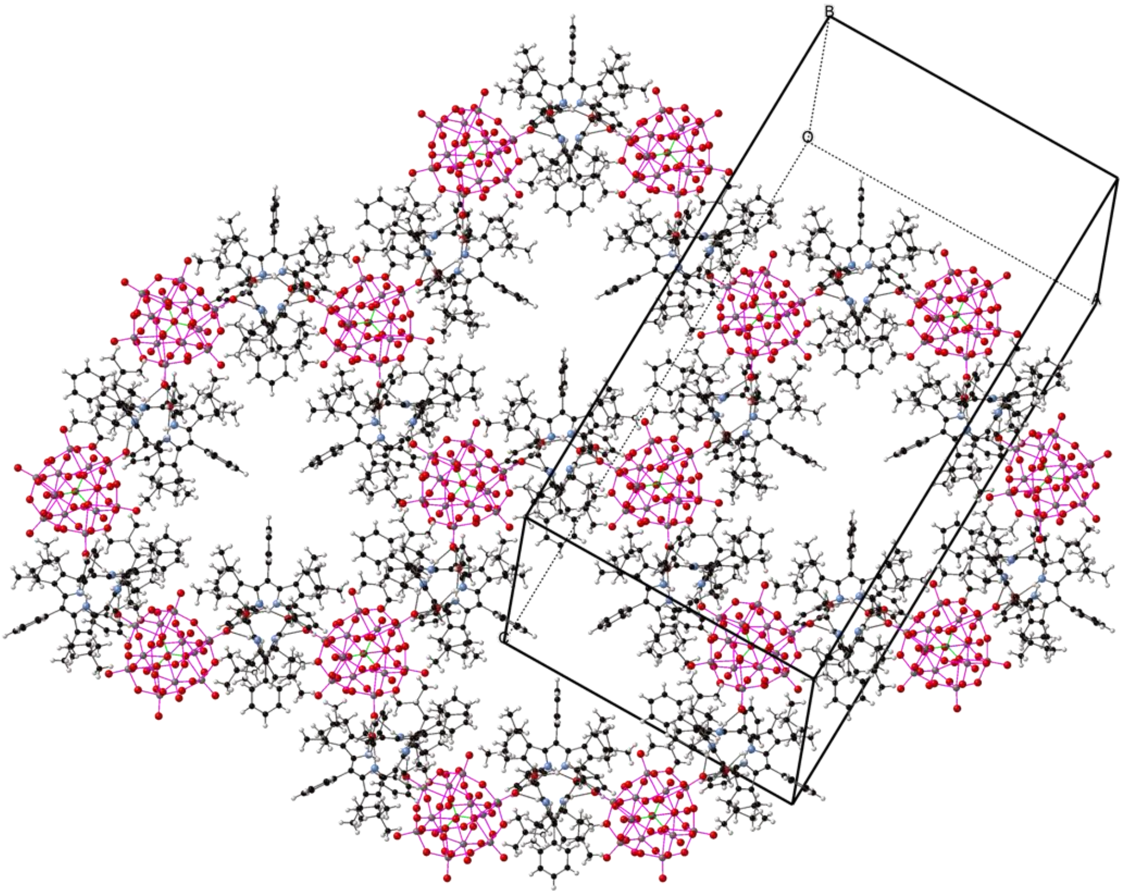

Figure S4.4; The (6,3-) net structure of $[H_4OiBuTPP]_3(Mo_{12}O_{40})_2(MeOH)_{55}(H_2O)_{1.5}$; voids within this structure were filled with a disordered solvent, which was quantified by use of Squeeze – the values obtained agreed broadly with methanol solvate – 195 molec./cell by volume, 216 molec./cell by electron density. 50 units per formula unit were assigned.

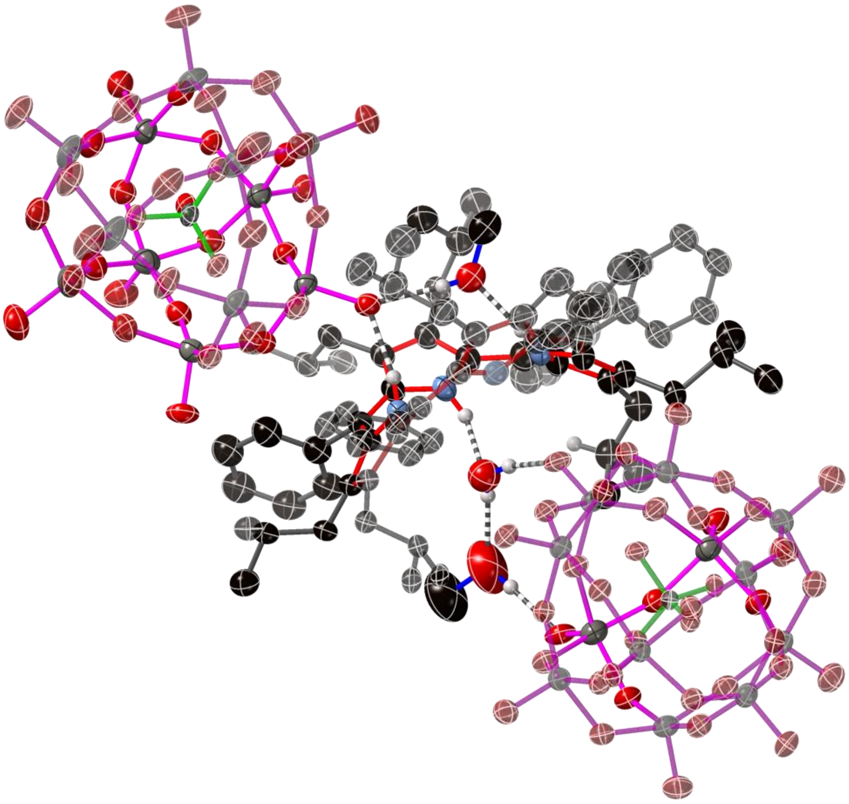

Figure S4.5; an example of the porphyrin core (red bonds) interacting with polyoxometalate (magenta bonds) and solvates (blue bonds)

S5; Supplementary images for Compound 6

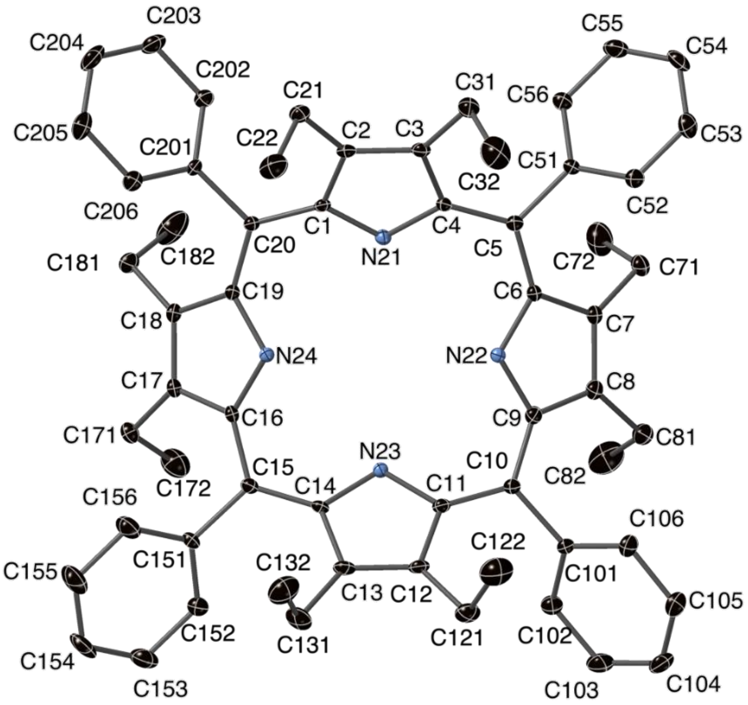

Figure S5.1; Labelled diagram of the porphyrin core in $[H_4OETPP](HSeO_3)_2(MeOSeO_2H)(MeOH)_3$; ellipsoids at 50%, H-atoms omitted.

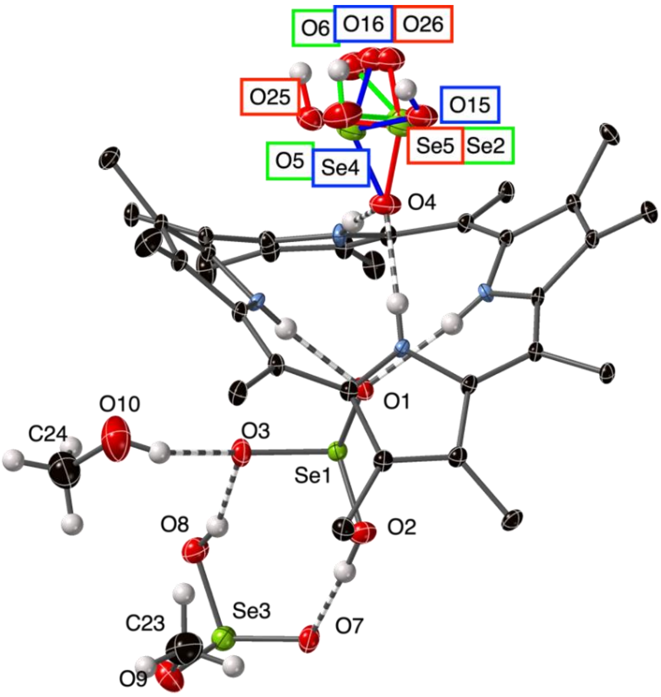

Figure S5.2; Hydrogen bonding and atom labels for the selenite and solvate components; the disorder of one hydrogenselenite anion into Se2 (0.674(2) occ., green), Se4 (0.0464(10) occ., blue) and Se5 (0.280(2) occ., red) is shown. Aryl and ethyl groups of the porphyrin core are omitted past the first atom. Hydrogen atoms on $MeOSeO_2H$ and SeO_3H^- are disordered over the two positions, and have lengths generally indicating 'symmetrical' hydrogen bonding.

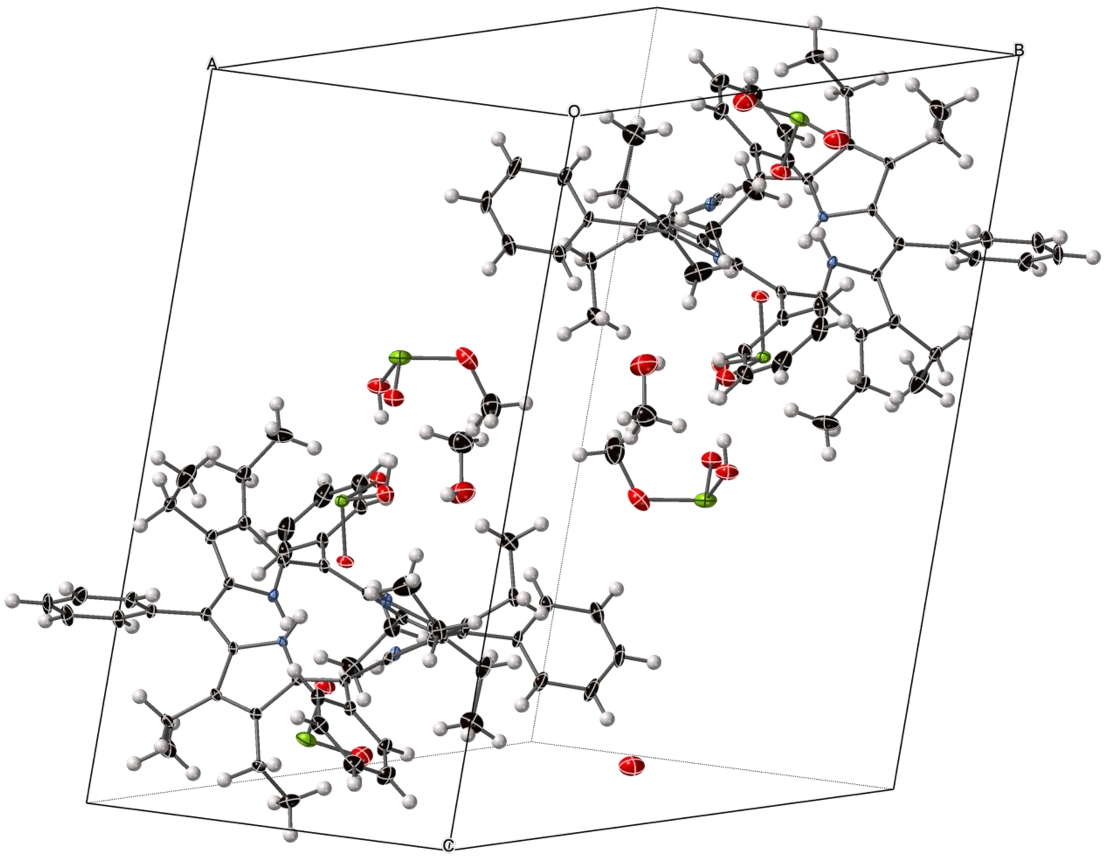

Figure S5.3; The atoms within the unit cell of $[H_4OETPP](HSeO_3)_2(MeOSeO_2H)(MeOH)_3$; Two of the methanol solvates in the formula unit are assigned from Squeeze results and therefore not shown.

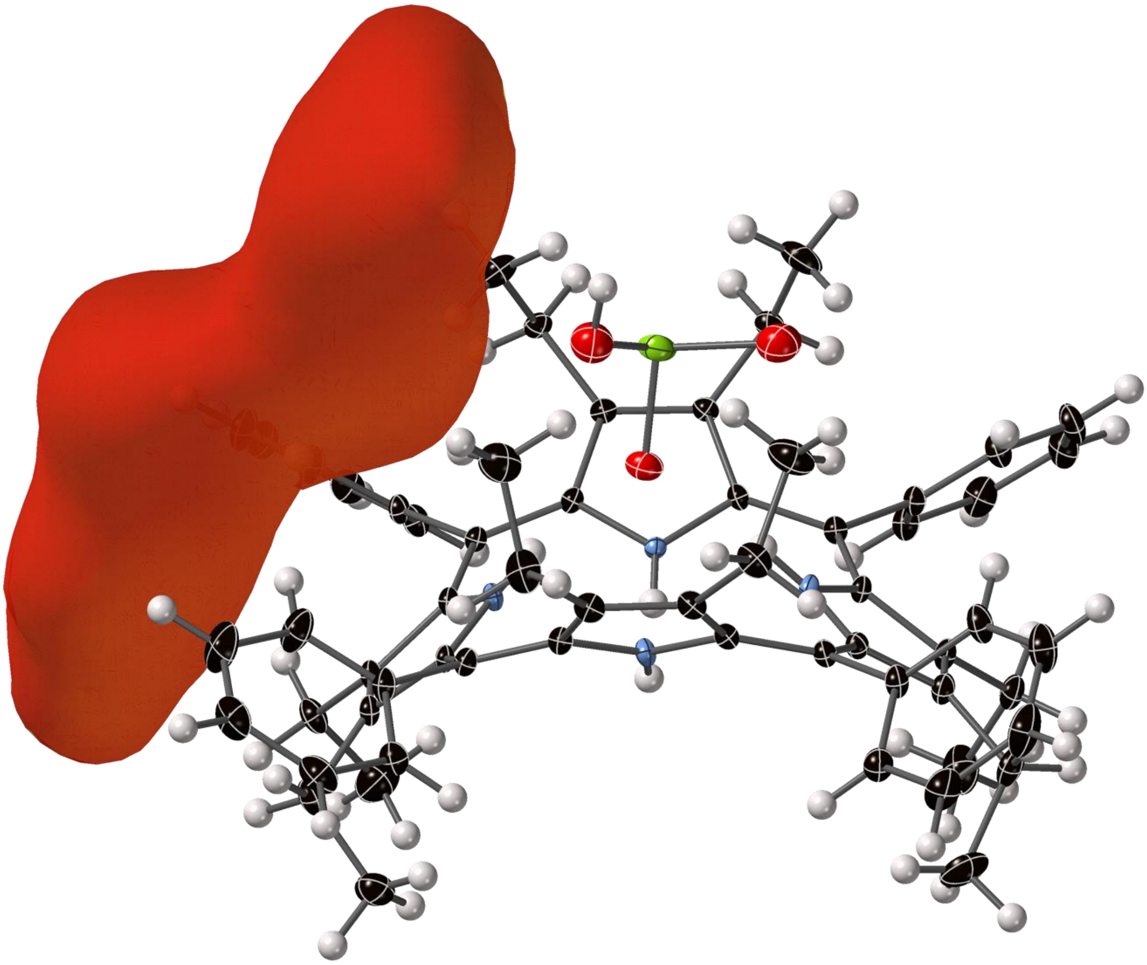

Figure S5.4; The solvent-filled void in $[H_4OETPP](HSeO_3)_2(MeOSeO_2H)(MeOH)_3$; two methanol molecules were assigned per formula unit based on Squeeze-indicated electron density.

S6; Supplementary images for Compound 7

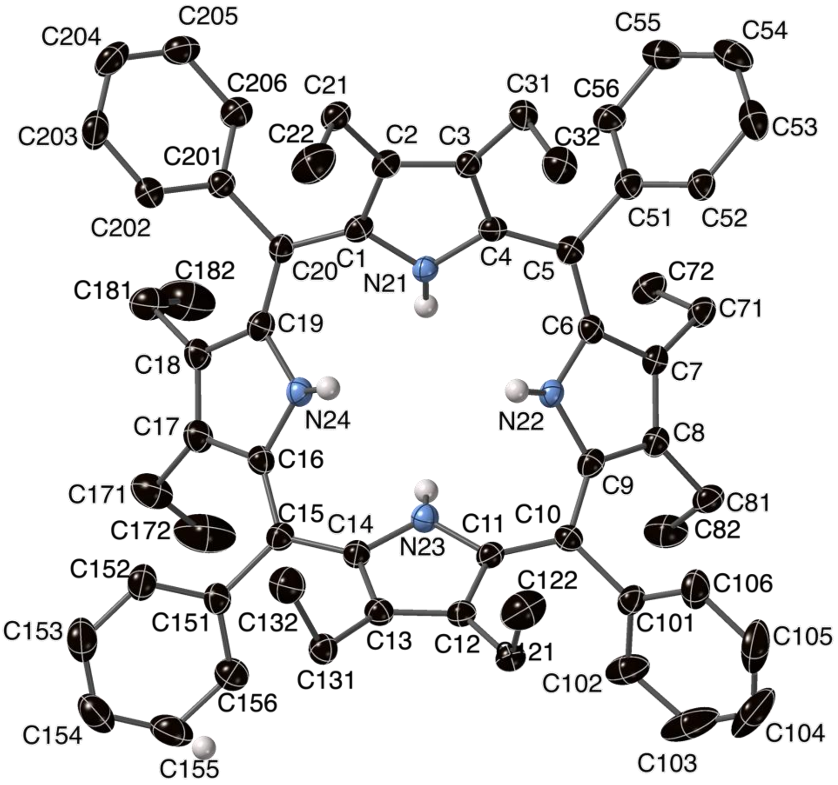

Figure S6.1; Labelled diagram of the porphyrin core in [H_4OETPP]($O_2C(CH_2)_{10}CO_2$)($MeOH$)$_4$(H_2O); C-bound H atoms omitted; Ellipsoids at 50%.

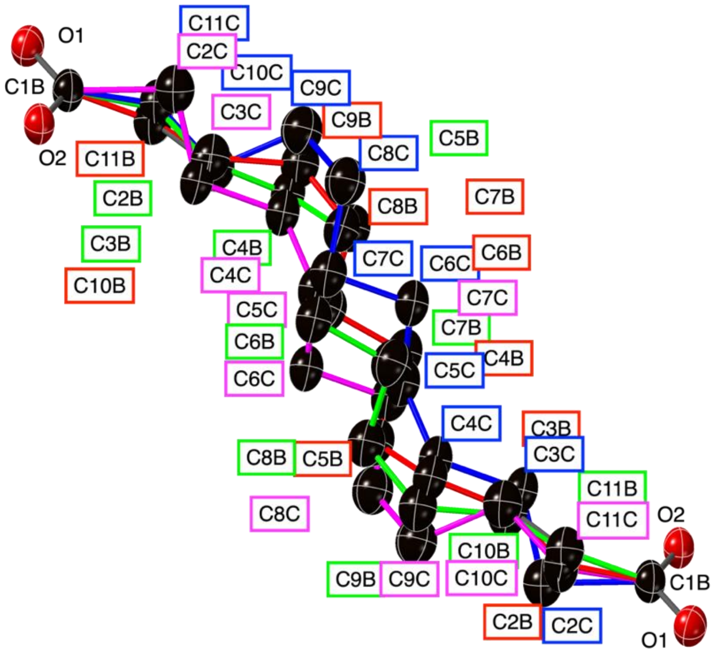

Figure S6.2; The four disordered orientations of one of the dodecandioate anions; This disordered structure exists on a centre of inversion, such that red and green are related by inversion, as are purple and blue. Many restraints and high thermal behaviour limit the insight on this anion beyond positive identification.

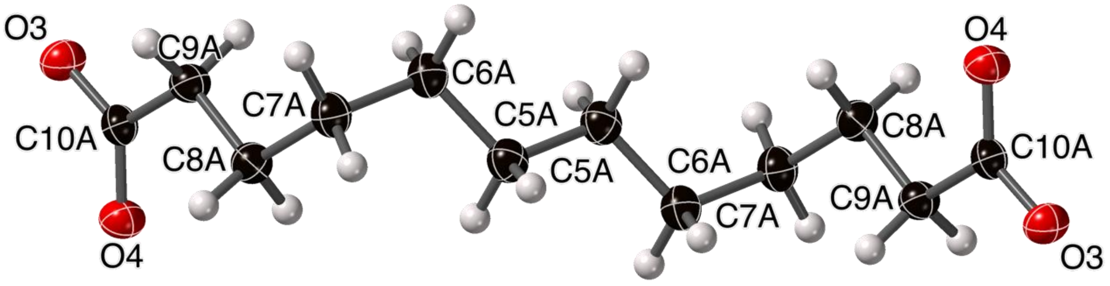

Figure S6.3; The second, ordered counteranion linking the porphyrin components; this anion is related to itself by a centre of inversion.

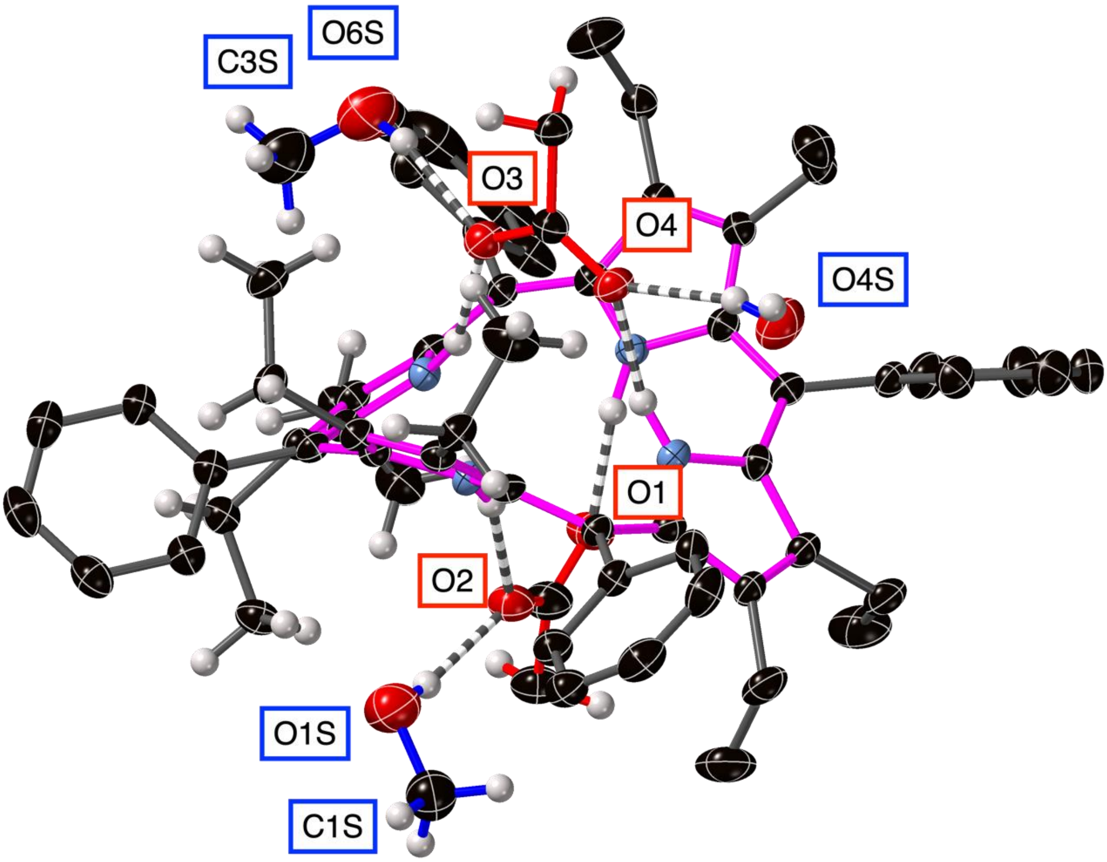

Figure S6.4; hydrogen bonding around the core of the porphyrin in $[H_4OETPP](O_2C(CH_2)_{10}CO_2)(MeOH)_4(H_2O)$; the core (purple bonds) N–H atoms interact with counteranions (red bonds) which also accept H-bonding interactions from methanol and water solvates (blue bonds).

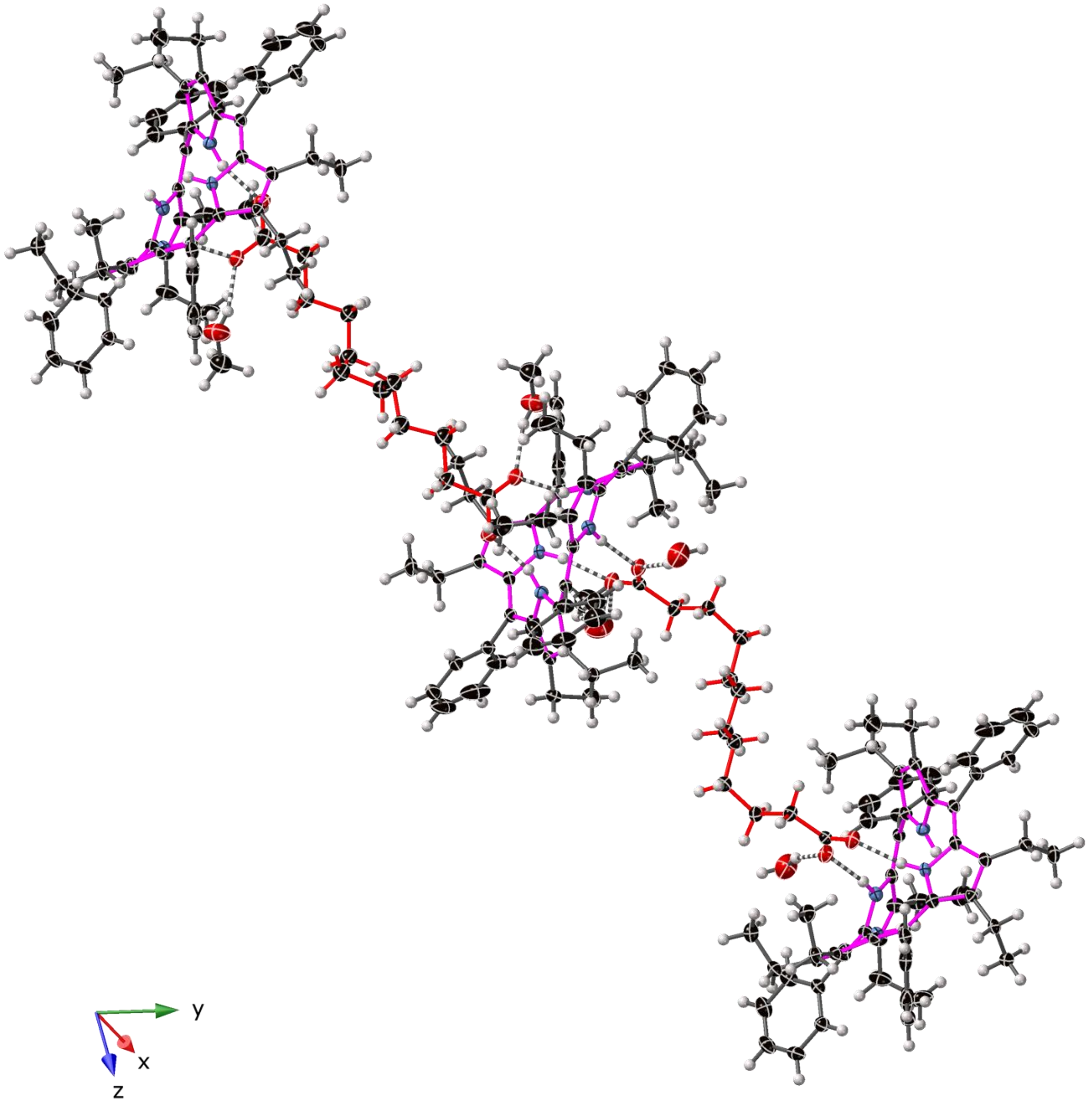

Figure S6.5; A short segment of the infinite chain of hydrogen bonding interactions which link porphyrin units into a one-dimensional H-bonded polymer

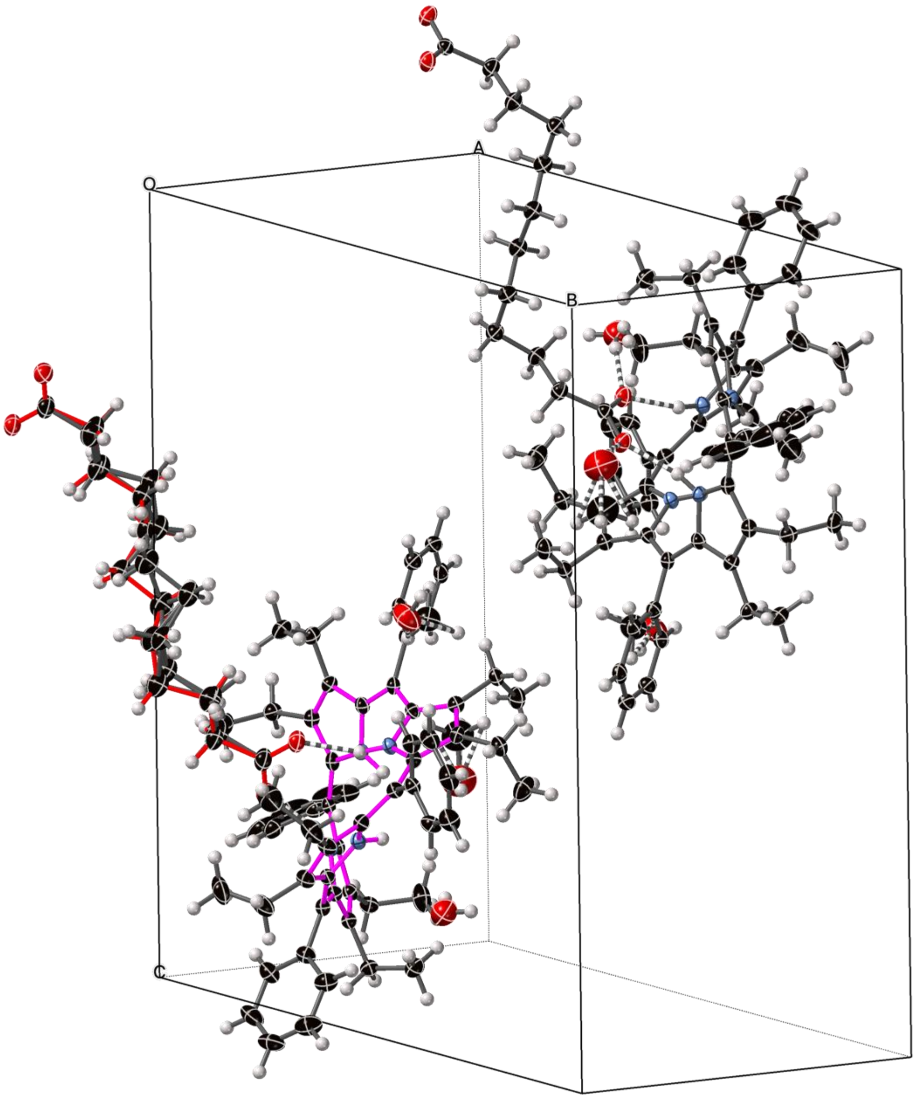

Figure S6.6; The molecules within a unit cell of [H_4OETPP]($O_2C(CH_2)_{10}CO_2$)(MeOH)$_4$(H_2O) (Z = 2) One porphyrin core (purple) and one anion orientation (red) are highlighted.

S7; Supplementary images for Compound 8

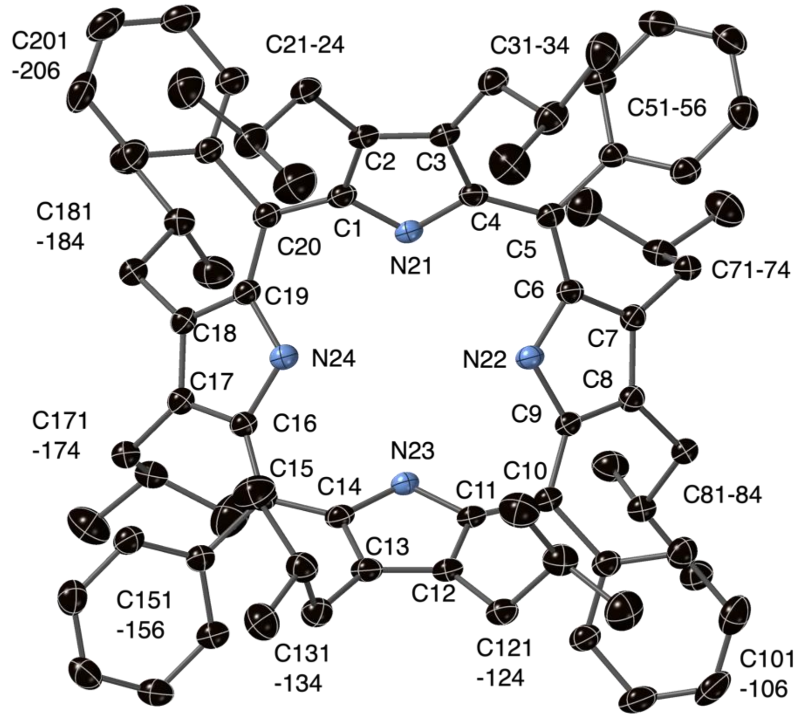

Figure S7.1; The labelled groups of the porphyrin component of [H_4OiBuTPP]($O_2CCH_2CO_2H$)(Cl)$_{0.7}$(Br)$_{0.3}$(MeOH)$_2$. Ellipsoids at 50%, H-atoms omitted.

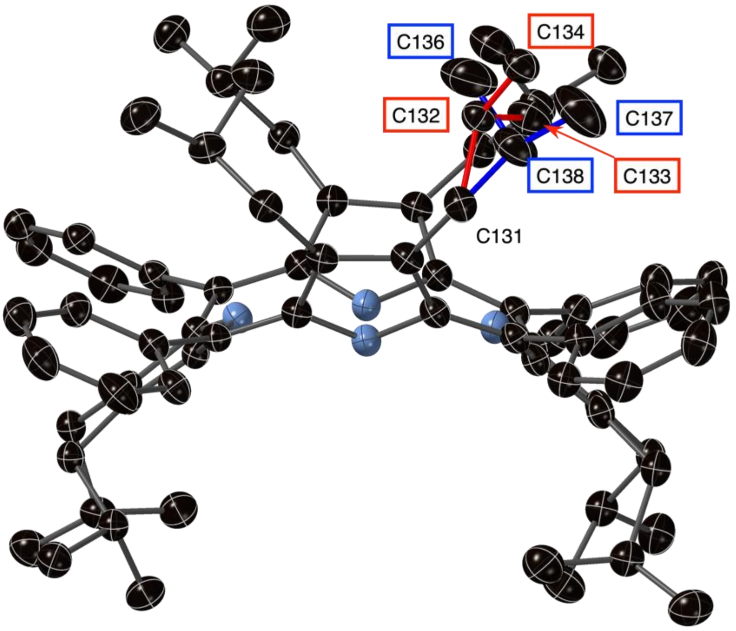

Figure S7.2; disorder of an iso-Butyl group over two orientations in the main fragment of the porphyrin. The two identified orientations are C131-134 (red, 0.689(6) occ.) and C135(n.s.)-138 (blue, 0.311(6) occ.). C131 and C135 were fixed to equal position and thermal parameters.

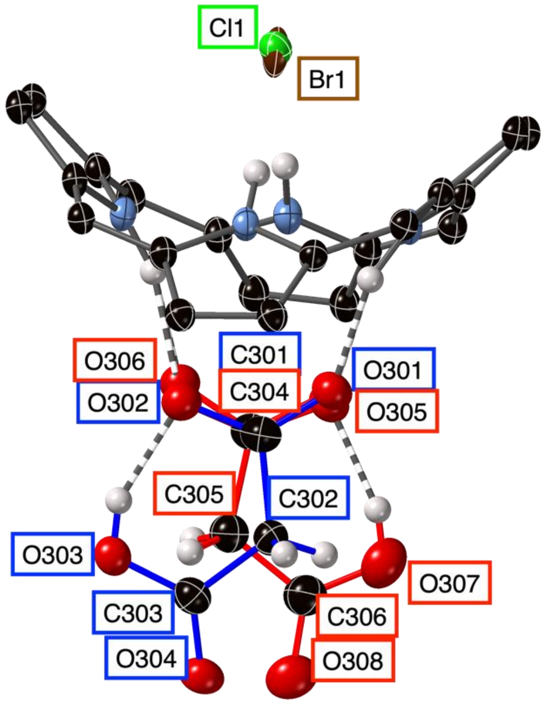

Figure S7.3; interactions with anions from the porphyrin core. The malonate mono-anion is disordered over two orientations, each involving an internal hydrogen bond; blue (O301-O304, C301-303, 0.821(3) occ.) and red (O305-308, C304-306, 0.179(3) occ.)

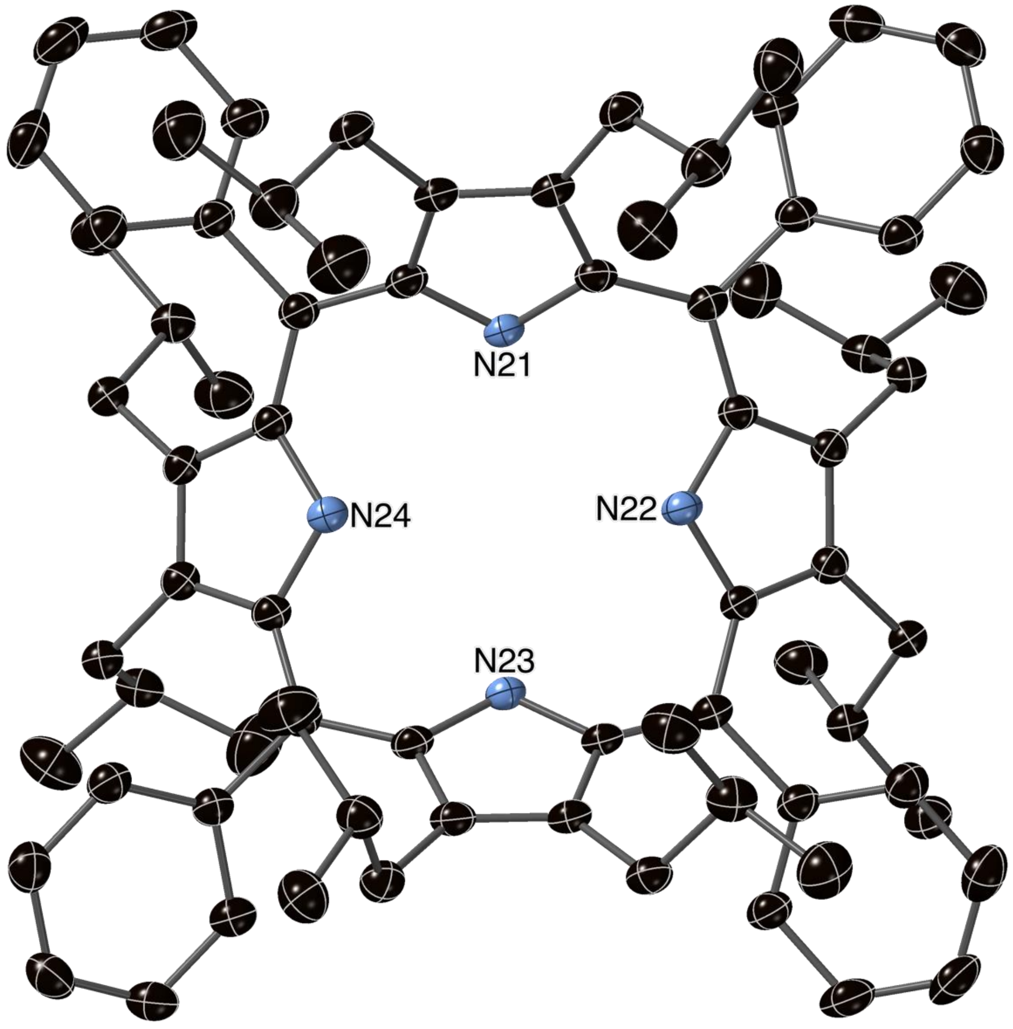

Figure S7.4; The porphyrin core with only N atoms labelled.

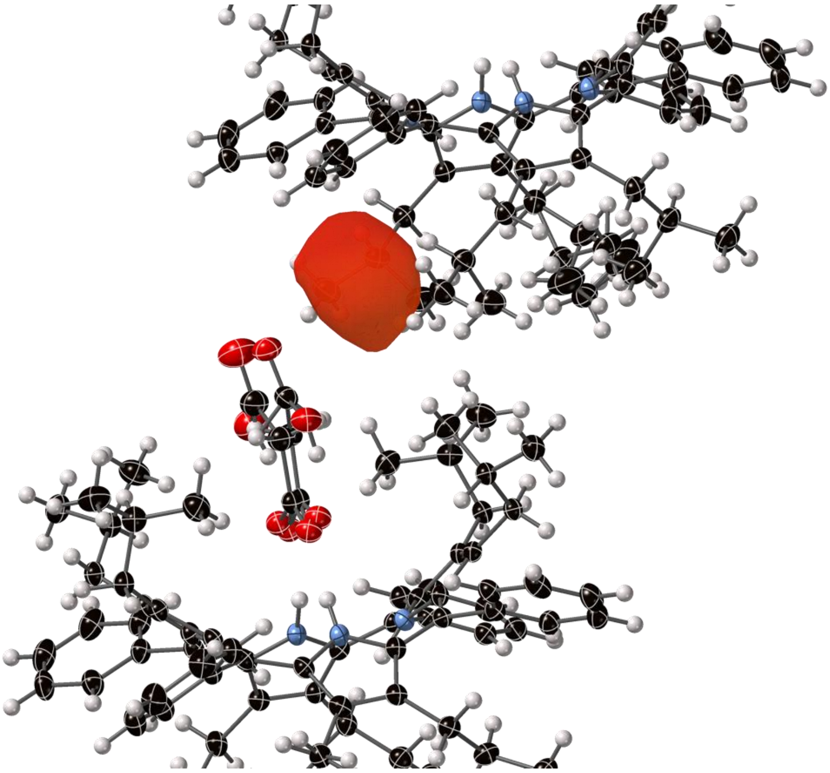

Figure S7.5; The solvent-filled void within the structure of **8**. No satisfactory model could be made of the solvent molecules.

S8; Supplementary images for Compound 9

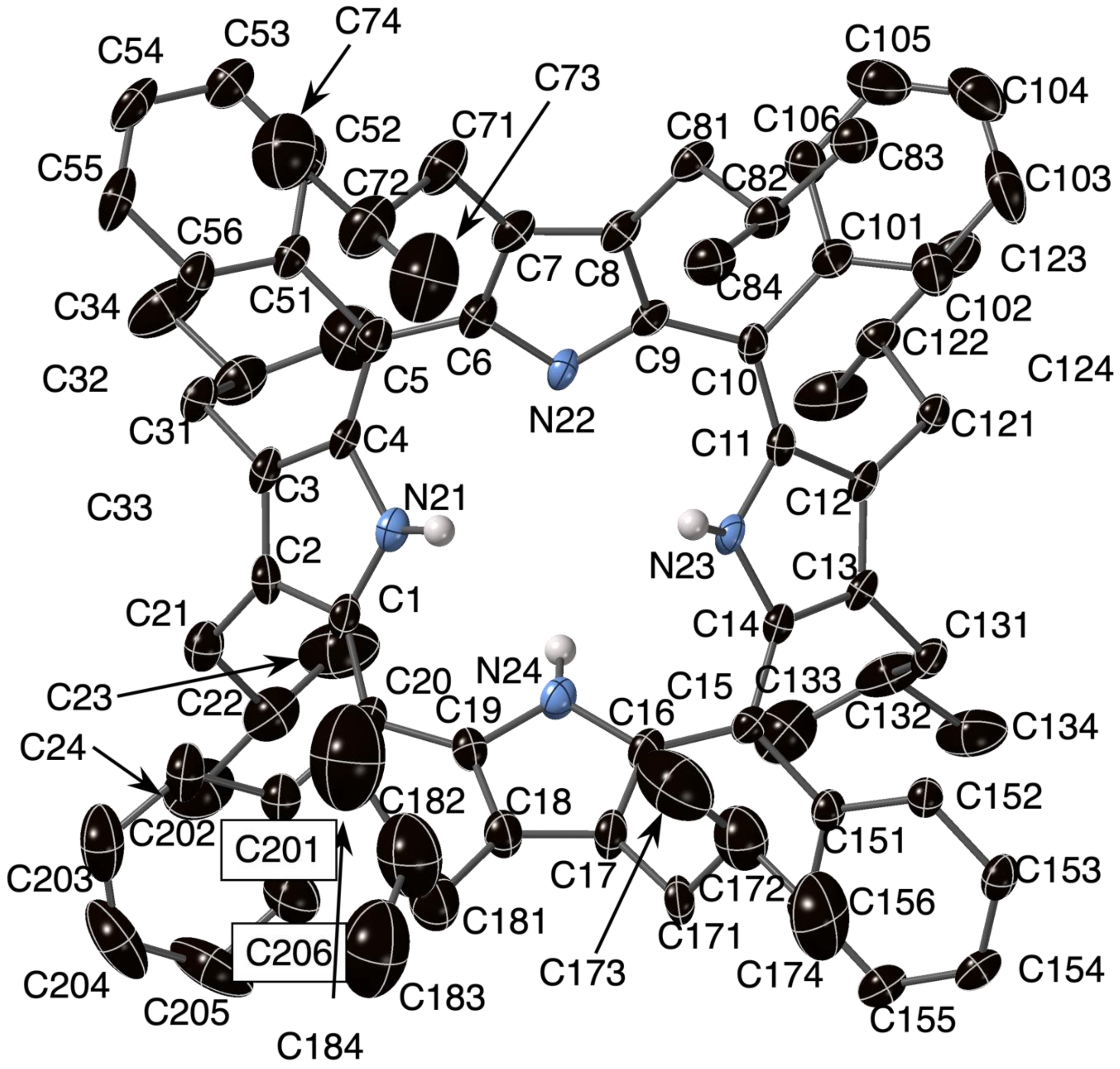

Figure S8.1; Labelled diagram of the porphyrin core in [H$_3$OETPP]$_2$(Mo$_4$O$_{12}$(OMe)$_2$)·6(MeOH); C-bound H atoms omitted; Ellipsoids at 50%.

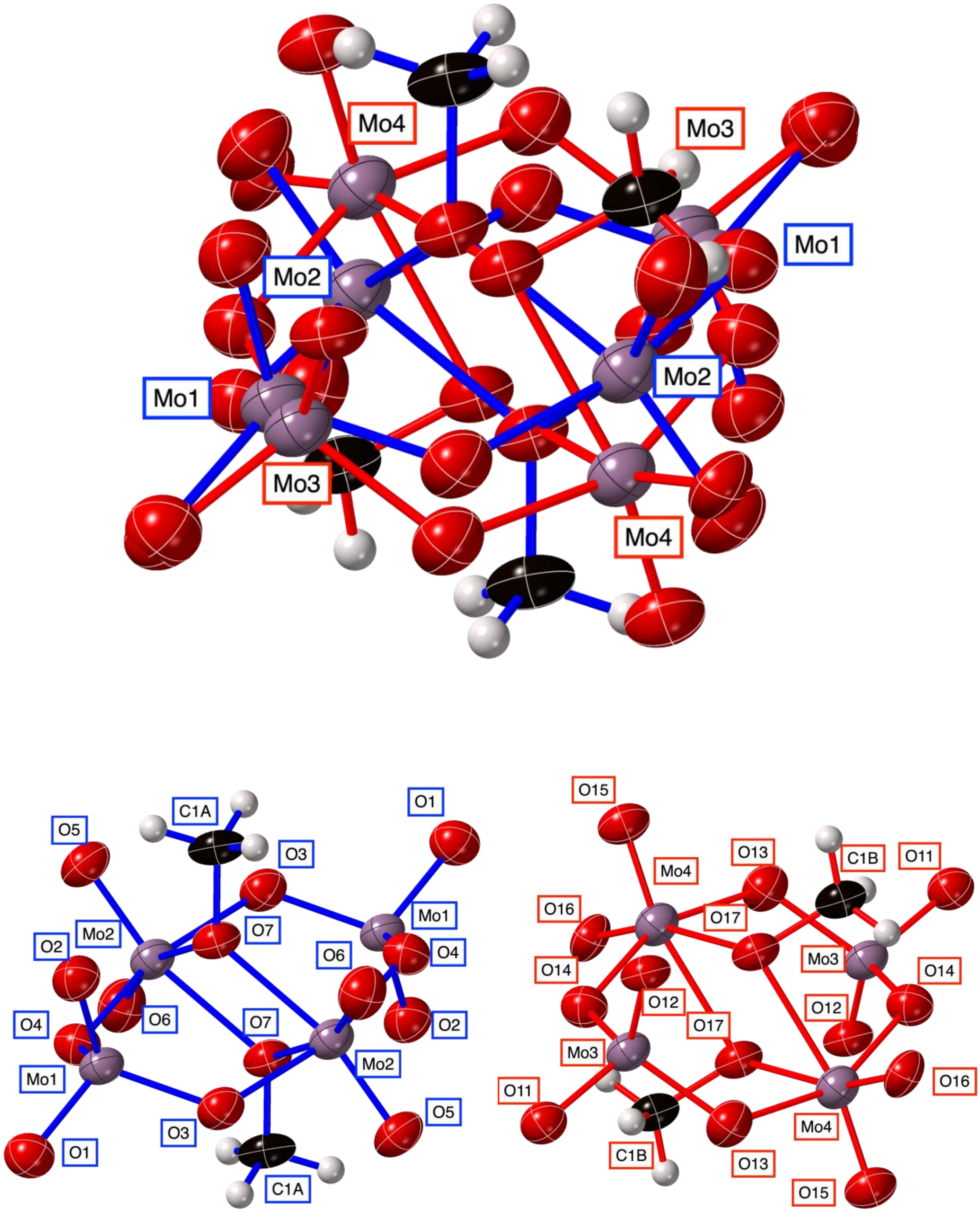

Figure S8.2; (a) the overlaid two disorder components of the tetramolybdate cluster, with Mo atoms labelled. (b and c) the disorder components (blue, Mo1-2/O1-7/C1A, 0.9486(11) occ. and red, Mo3-4/O11-17/C1B occ. 0.0514(11)) shown separately and labelled. This cluster featuring two tetrahedral and two octahedral Mo atoms has seemingly not been previously crystallographically identified; the related $[Mo_4O_{10}(OMe)_6]$ https://doi.org/10.1016/S0277-5387(00)87151-X shares similar features of charge, ligands, and number of metal centers.

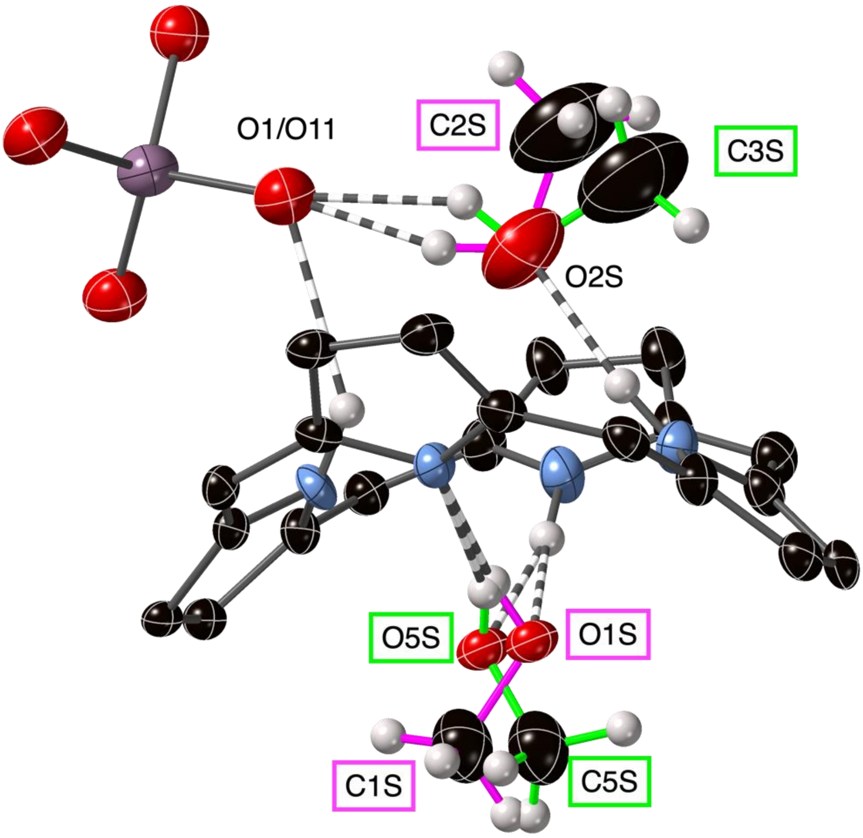

Figure S8.3; Disorder within the identified solvates of $[H_3OETPP]_2(Mo_4O_{12}(OMe)_2)\cdot 6(MeOH)$. Methanol over two sites O1S (magenta, 0.788(14) occ.) and O5S (green, 0.212(14) occ.) are both accepting and donating H-bonds to the porphyrin core. O2S engages in a two-site interaction between the tetramolybdate anion and a solvate. Internal angles of the porphyrin unit indicate that the donor NH is localized to this position.

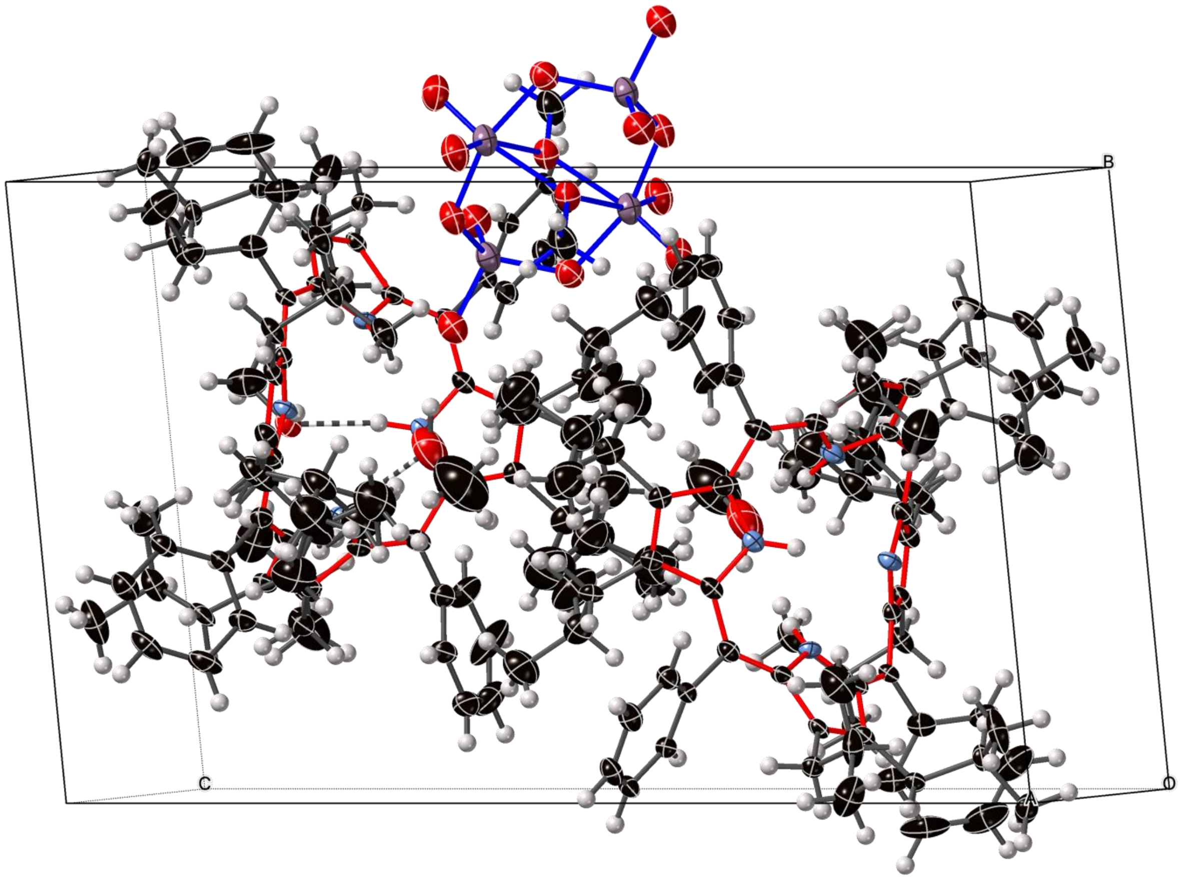

Figure S8.4; Molecules within the unit cell; the molybdate (blue) and porphyrin (red) are highlighted.

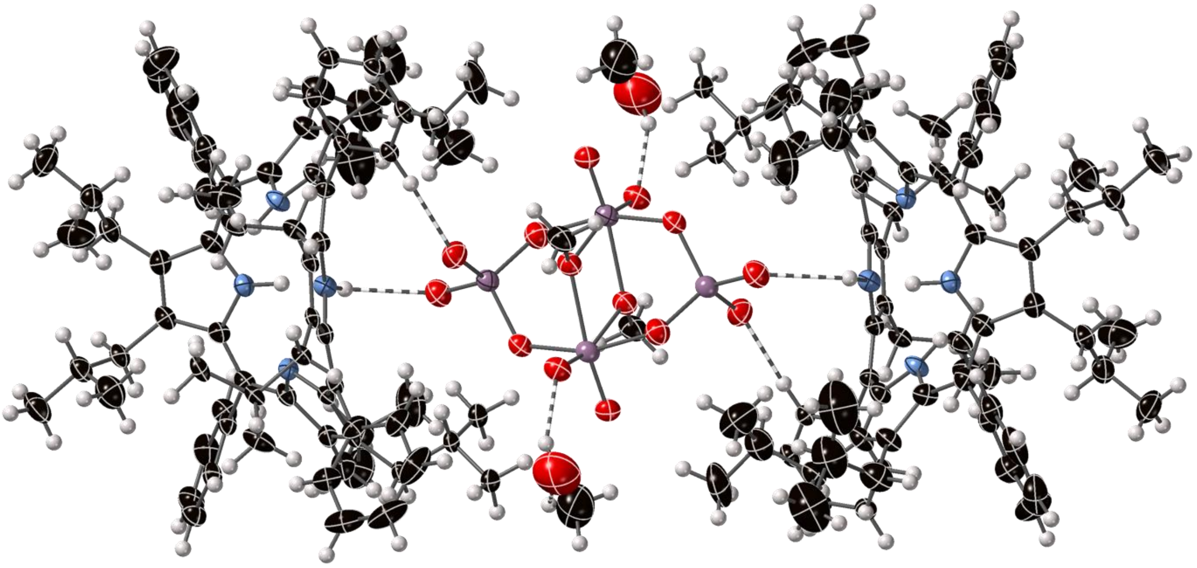

Figure S8.5; the H-bonding and C–H···O interactions through which two porphyrins are linked by an anion.

S9; Supplementary images for Compound 10

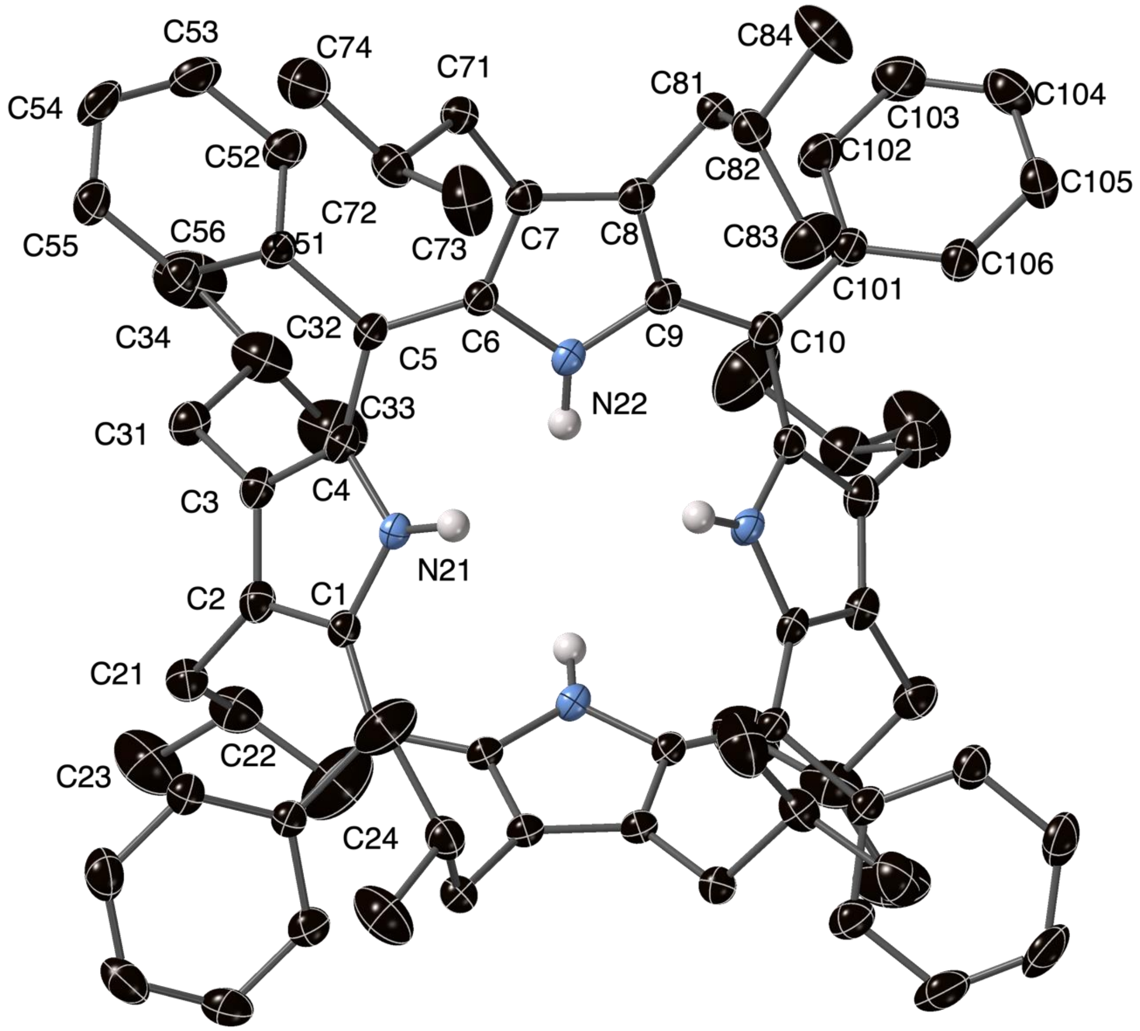

Figure S9.1; Labelled diagram of the porphyrin core in $[H_3O(iBu)TPP](OAc)(MeOH)_{0.308}(H_2O)_{0.692}(DCM)_{1.25}$; C-bound H atoms omitted; Ellipsoids at 50%. A second orientation of C21-24 and C31-34 is omitted.

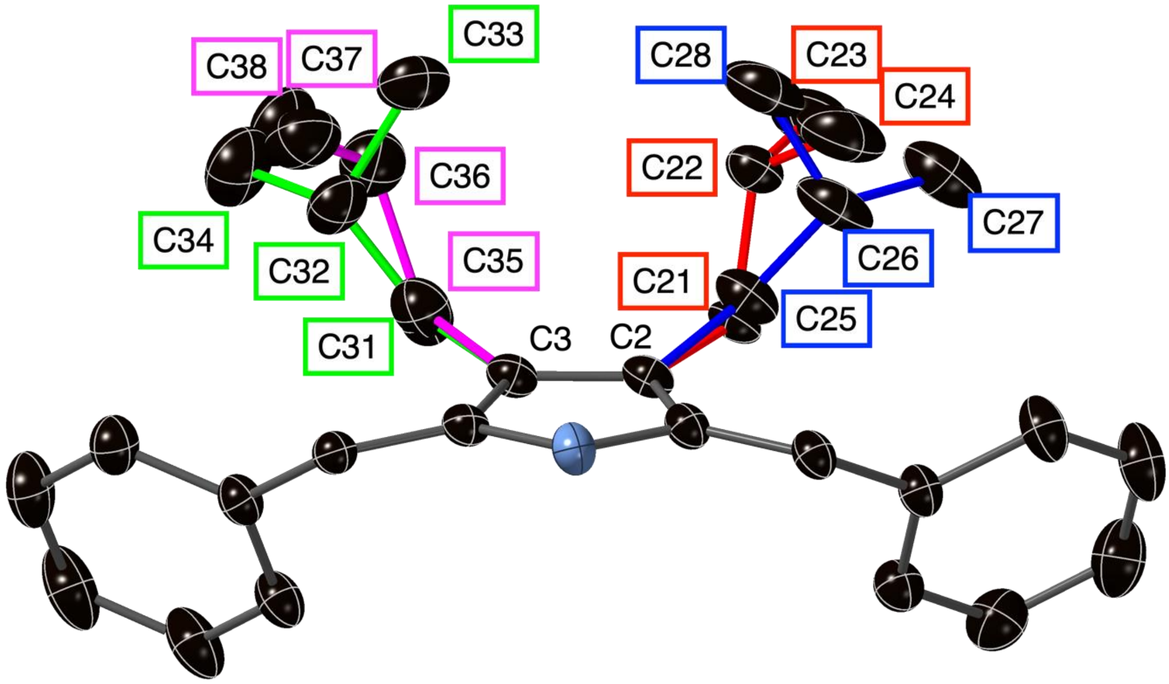

Figure S9.2; disorder within the side-chains of the $[H_3O(iBu)TPP]^+$ monocation. C21-24 (red, 0.854(4) occ.) C25-28 (blue, 0.146(4) occ.) C31-34 (green, 0.827(5) occ.) and C35-38 (magenta, 0.173(5)).

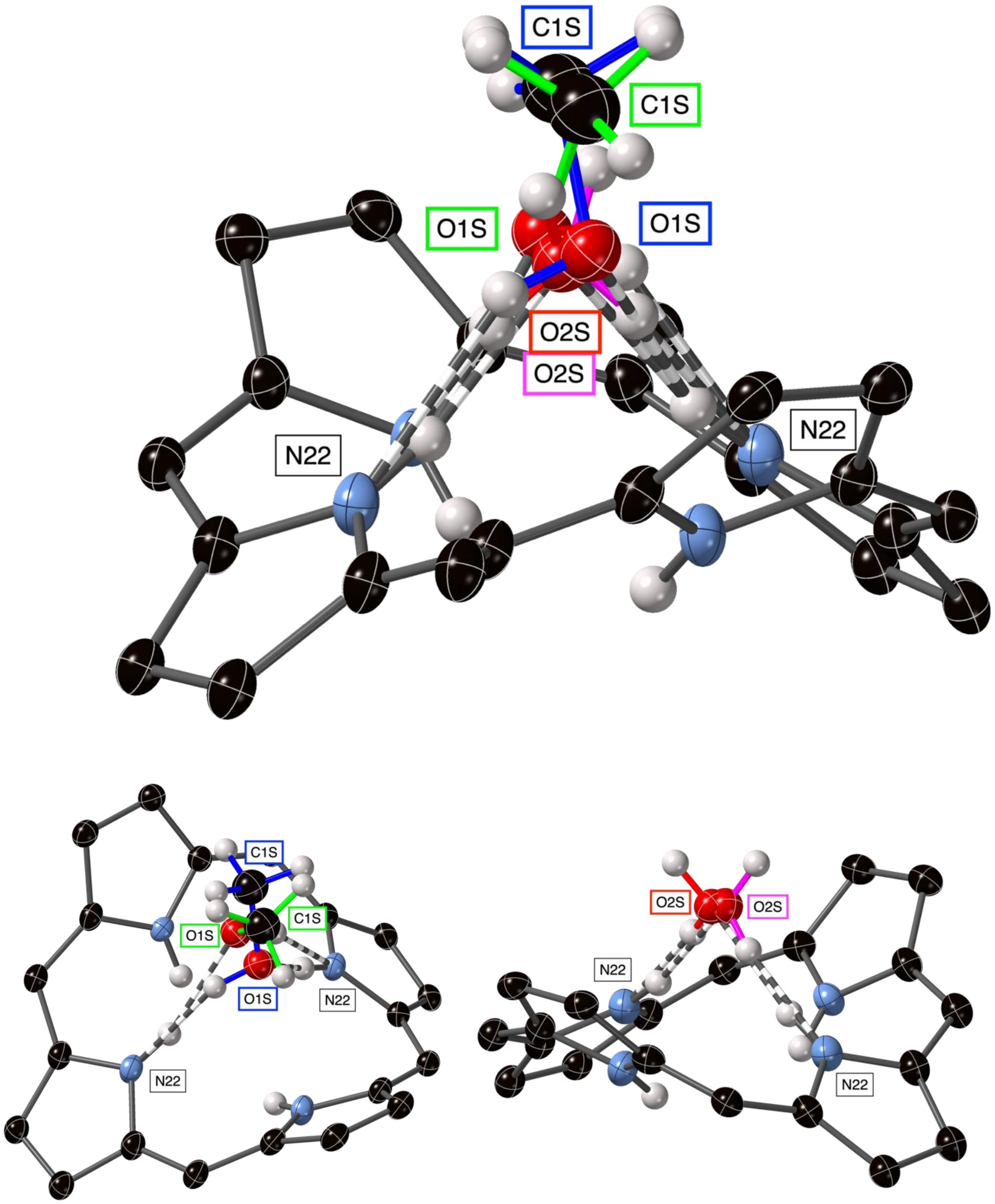

Figure S9.3; the disordered solvent site interacting with N22; (a) all components (sum occ. = 1) (b) the methanol unit, disordered over two equivalent orientations, sum occ. 0.308 (c) the water units, disordered over two equivalent orientations, sum occ. 0.692. Each component acts as H-bond donor and acceptor each to one of the equivalent N22 sites, thus the occupancy of H22 is fixed at 0.5.

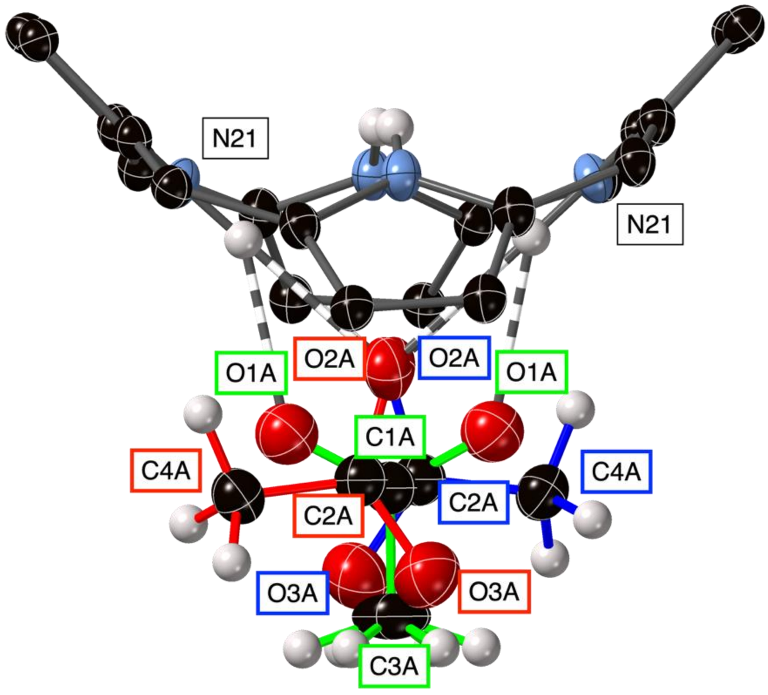

Figure S9.4; disorder within the acetate anion which is bound to the N21 atoms of the porphyrin core; the two equivalent 'side-on' orientations (O2A, O3A, C2A, C4A; red and blue) are 0.215(3) occ. each, with the 'front-on' orientation (O1A, C1A, C3A) is occupied at 0.571(6).

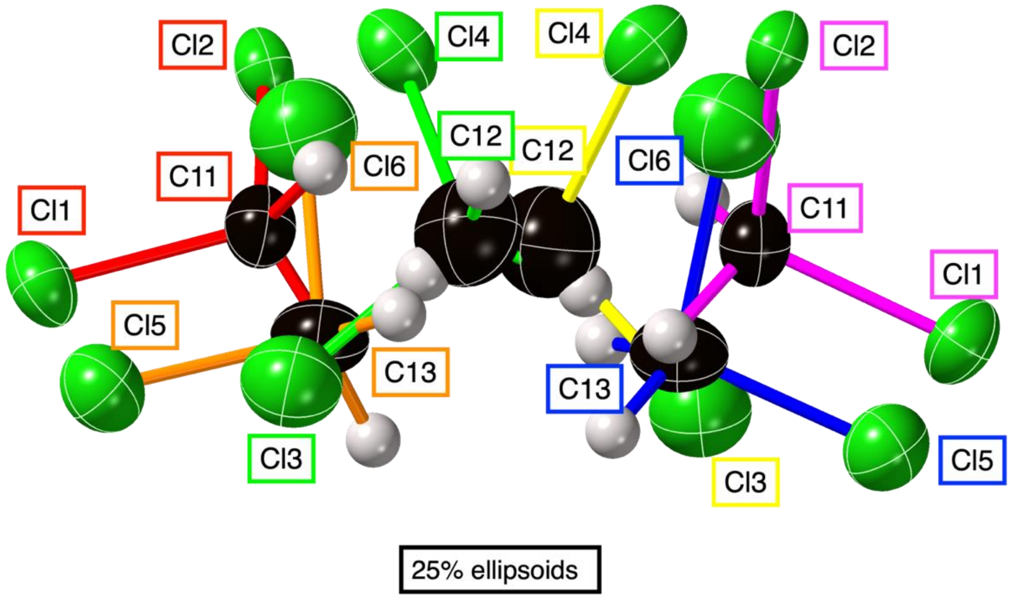

Figure S9.5; disordered dichloromethane in the structure of **10**; the disordered solvate pocket contains 1.25 chloroform units. C11/Cl1/Cl2 (red and magenta, 0.357(2) occ.) C12/Cl3/Cl4 (green and yellow, 0.1695(19) occ.) and C13/Cl5/Cl6 (orange and blue, 0.098(2) occ.)

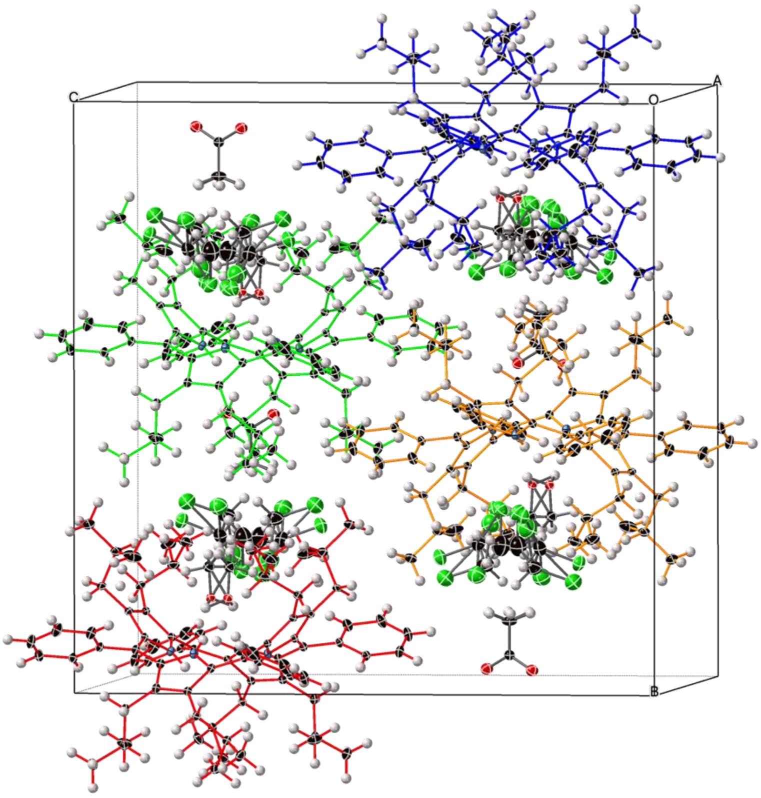

Figure S9.6; The molecules within the unit cell of **10**, with the four equivalent porphyrin units highlighted in red, orange, green and blue. Minor components of disorder are omitted.

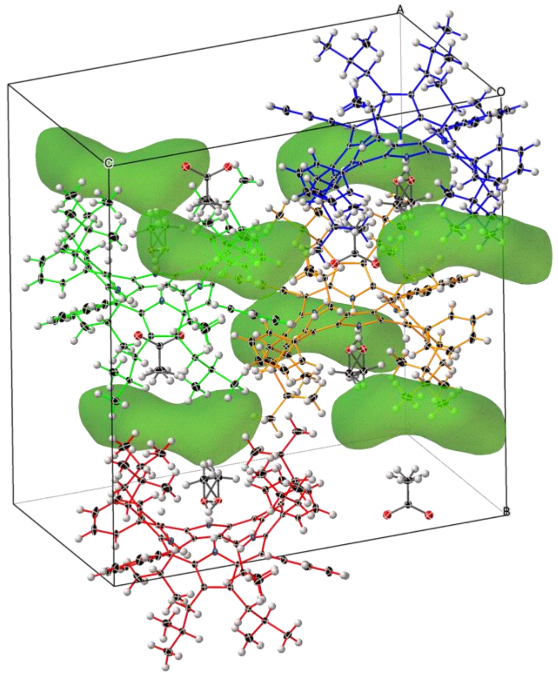

Figure S9.7; Pockets of disordered dichloromethane (green surfaces) within the structure of 10.

S10; Supplementary images for Compound 11

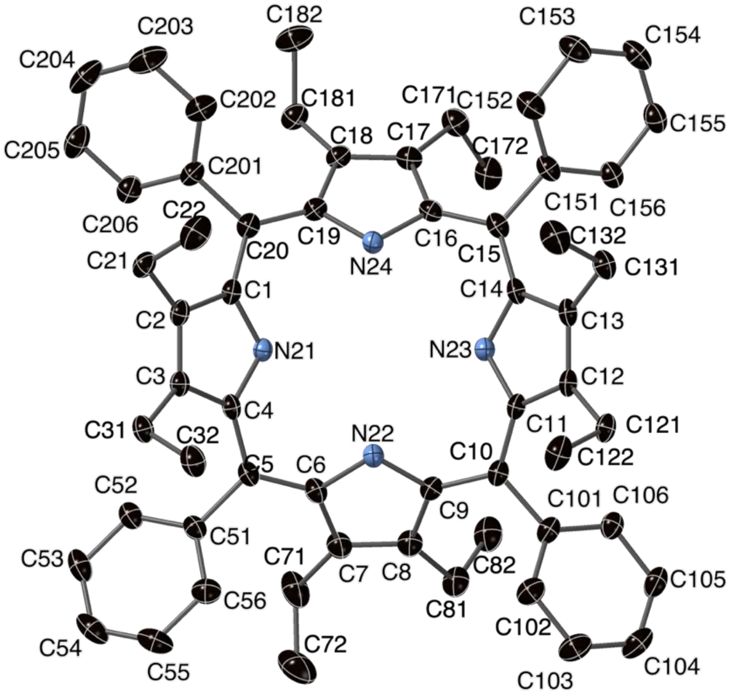

Figure S10.1; Labelled structure of $[H_4OETPP]^{2+}$ within $[H_4OETPP](m\text{-}(CO_2H)C_6H_4COO^-)_2\ (MeOH)_{2.6}(DCM)_{0.55}$

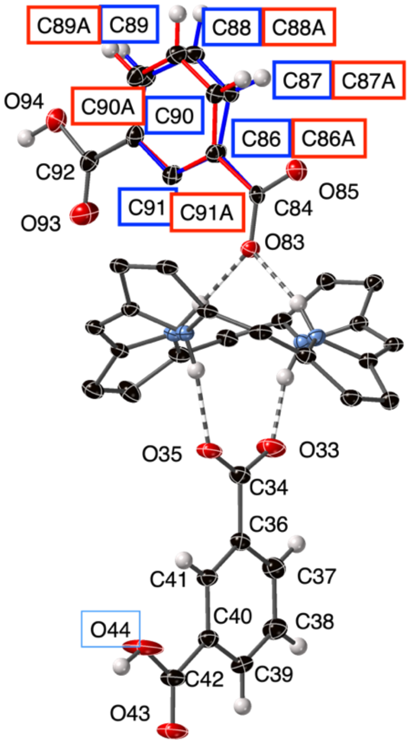

Figure S10.2; Interactions between two phthalate monoanions and the porphyrin core; the ring of one phthalate is disordered over two orientations; C86-91 (blue, 0.67 occ.) and C86A-91A (red, 0.33 occ.).

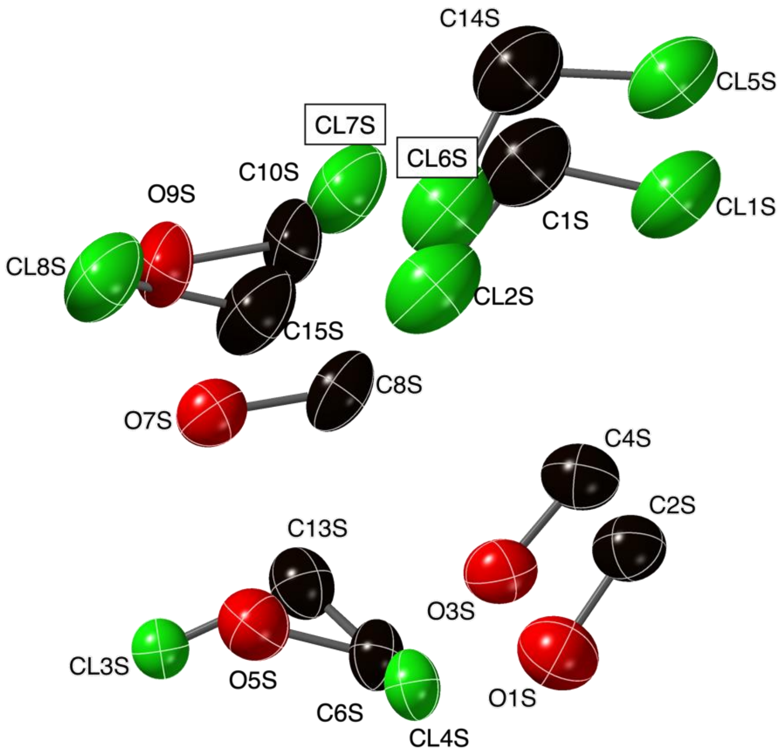

Figure S10.3; thermal ellipsoids of the different solvate units in space between porphyrins.

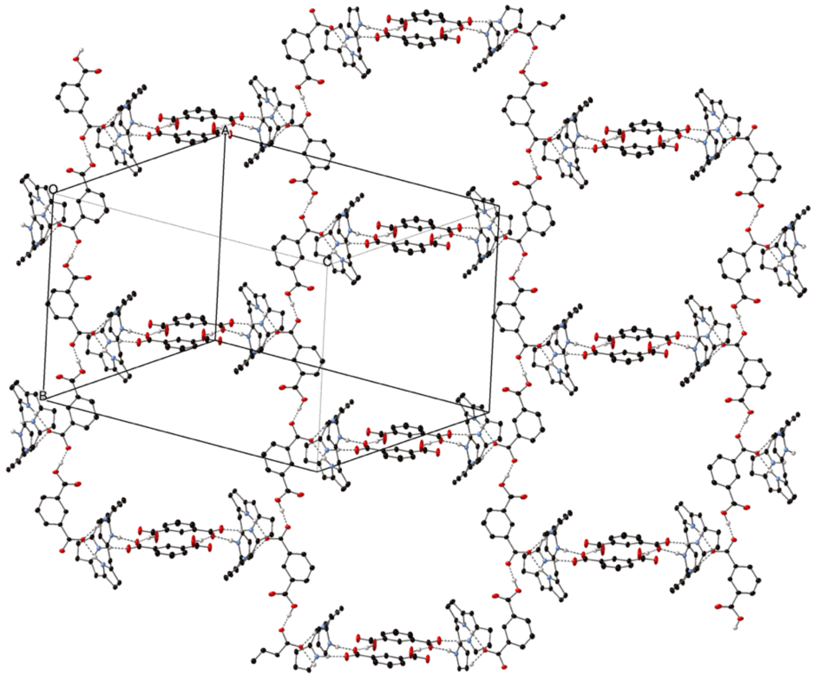

Figure S10.4; The hydrogen-bonding network which is formed by the combination of the porphyrin core H-bonds and those of the hydrogenphthalate; this resembles a honeycomb arrangement of the porphyrin components, linked through singular or double phthalate H-bond linkages.
